# Knock-in and precise nucleotide substitution using near-PAMless engineered Cas9 variants in *Dictyostelium discoideum*

**DOI:** 10.1038/s41598-021-89546-0

**Published:** 2021-05-27

**Authors:** Yuu Asano, Kensuke Yamashita, Aoi Hasegawa, Takanori Ogasawara, Hoshie Iriki, Tetsuya Muramoto

**Affiliations:** Department of Biology, Faculty of Science, Toho University, 2-2-1 Miyama, Funabashi, Chiba 274-8510 Japan

**Keywords:** Biotechnology, Molecular biology

## Abstract

The powerful genome editing tool *Streptococcus pyogenes* Cas9 (SpCas9) requires the trinucleotide NGG as a protospacer adjacent motif (PAM). The PAM requirement is limitation for precise genome editing such as single amino-acid substitutions and knock-ins at specific genomic loci since it occurs in narrow editing window. Recently, SpCas9 variants (i.e., xCas9 3.7, SpCas9-NG, and SpRY) were developed that recognise the NG dinucleotide or almost any other PAM sequences in human cell lines. In this study, we evaluated these variants in *Dictyostelium discoideum*. In the context of targeted mutagenesis at an NG PAM site, we found that SpCas9-NG and SpRY were more efficient than xCas9 3.7. In the context of NA, NT, NG, and NC PAM sites, the editing efficiency of SpRY was approximately 60% at NR (R = A and G) but less than 22% at NY (Y = T and C). We successfully used SpRY to generate knock-ins at specific gene loci using donor DNA flanked by 60 bp homology arms. In addition, we achieved point mutations with efficiencies as high as 97.7%. This work provides tools that will significantly expand the gene loci that can be targeted for knock-out, knock-in, and precise point mutation in *D. discoideum*.

## Introduction

Under optimal nutrient conditions, the social amoeba *D. discoideum* grows as a single-cell organism; however, in response to starvation, about 100,000 cells aggregate to form a multicellular fruiting body that consists of stalk and spore cells. It possesses a rather small haploid genome (~ 34 Mb) that contains a wide range of homologous genes involved in cell motility, signal transduction, chemotaxis, phagocytosis, and multicellular formation^1,2^. The high efficiency of gene manipulations in this organism, including knock-out and knock-in by homologous recombination, RNA interference (RNAi), and overexpression, has enabled us to study the functions of these genes in detail^3–6^.

Recently, genome editing using the clustered regularly interspaced short palindromic repeats (CRISPR)-associated protein 9 (Cas9) has been developed as a toolbox for functional analysis in a variety of organisms^7–9^. In *D. discoideum*, all-in-one vectors capable of expressing Cas9 endonuclease and a chimeric single-guide RNA (sgRNA) that recognises target sequences have allowed highly efficient gene disruption^10,11^. Although one issue with CRISPR is off-target effects, these vectors are not stably maintained in cells, minimising the frequency of unwanted editing. Despite the transient expression of the CRISPR components, more than half of the cells were edited^11^, indicating that this system is both highly efficient and relatively specific. Furthermore, using the double-nicking method with Cas9 nickase, the probability of off-target effects can be further reduced, and target-specific genome deletions larger than 1 kb have been successfully introduced in *D. discoideum*^12^. Recruitment of Cas9 to target DNA is programmed by sgRNA, but Cas9 also requires a PAM flanking the target site^13–15^. NGG is the canonical PAM sequence for SpCas9, the endonuclease most widely used in CRISPR. The PAM requirement significantly decreases the number of targetable sites, especially in the AT-rich *D. discoideum* genome. Homology-directed repair (HDR) mediated gene knock-in and precise nucleotide substitution, which are genome editing applications that require high-resolution targeting, are even more strongly affected by the PAM requirement because these types of editing must generally occur in a narrow window around the target sequence.

To overcome these limitations on targeting range, several Cas9 variants with distinct PAM sequences have been developed, including SpCas9-VQR, SpCas9-VRER, and SpCas9-EQR^16^. In addition, Cas9 orthologs derived from other species with different PAM sequences, such as *Staphylococcus aureus* Cas9 (SaCas9), SaCas9 variant (SaCas9-KKH), *Streptococcus canis* (ScCas9), *Streptococcus thermophilus 1* (St1Cas9), *Streptococcus thermophilus 3* (St3Cas9), and *Campylobacter jejuni* Cas9 (CjCas9), have also been characterised^17–22^. Furthermore, the newly engineered SpCas9 variants, xCas9 3.7 and SpCas9-NG can recognise the non-canonical NGN PAM in human cells^23,24^, and have been successfully tested in rice, *Arabidopsis*, and mice^25–28^. Comparison of xCas9 3.7 and SpCas9-NG in mammalian cells and plants revealed that SpCas9-NG has a higher editing efficiency than xCas9 3.7 at NG PAMs^24,29,30^. More recently, a structure-based engineering approach was used to generate SpRY, which can edit almost all PAMs (NRN > NYN) in mammalian cells^29^. However, it remains unclear whether SpRY functions effectively in other model organisms, including *D. discoideum*. A demonstration that SpCas9 variants such as xCas9 3.7, SpCas9-NG, and SpRY could be used in *D. discoideum* would expand the potential for gene knockouts, gene knock-ins, and precise base substitutions in regions that do not contain canonical NGG PAMs.

In this study, we generated transient all-in-one vectors to evaluate the editing efficiencies of SpCas9, xCas9 3.7, SpCas9-NG, and SpRY at various PAM sites in *D. discoideum*. All of the Cas9 variants could edit target regions harbouring NG PAM sequences. In addition, we showed that SpRY had robust nuclease activity at almost all PAMs. Moreover, SpRY improved editing resolution at precise positions for gene knock-in and nucleotide substitution.

## Results

### Cas9 variant-mediated genome editing in tdTomato knock-in cells

**Figure 1:**
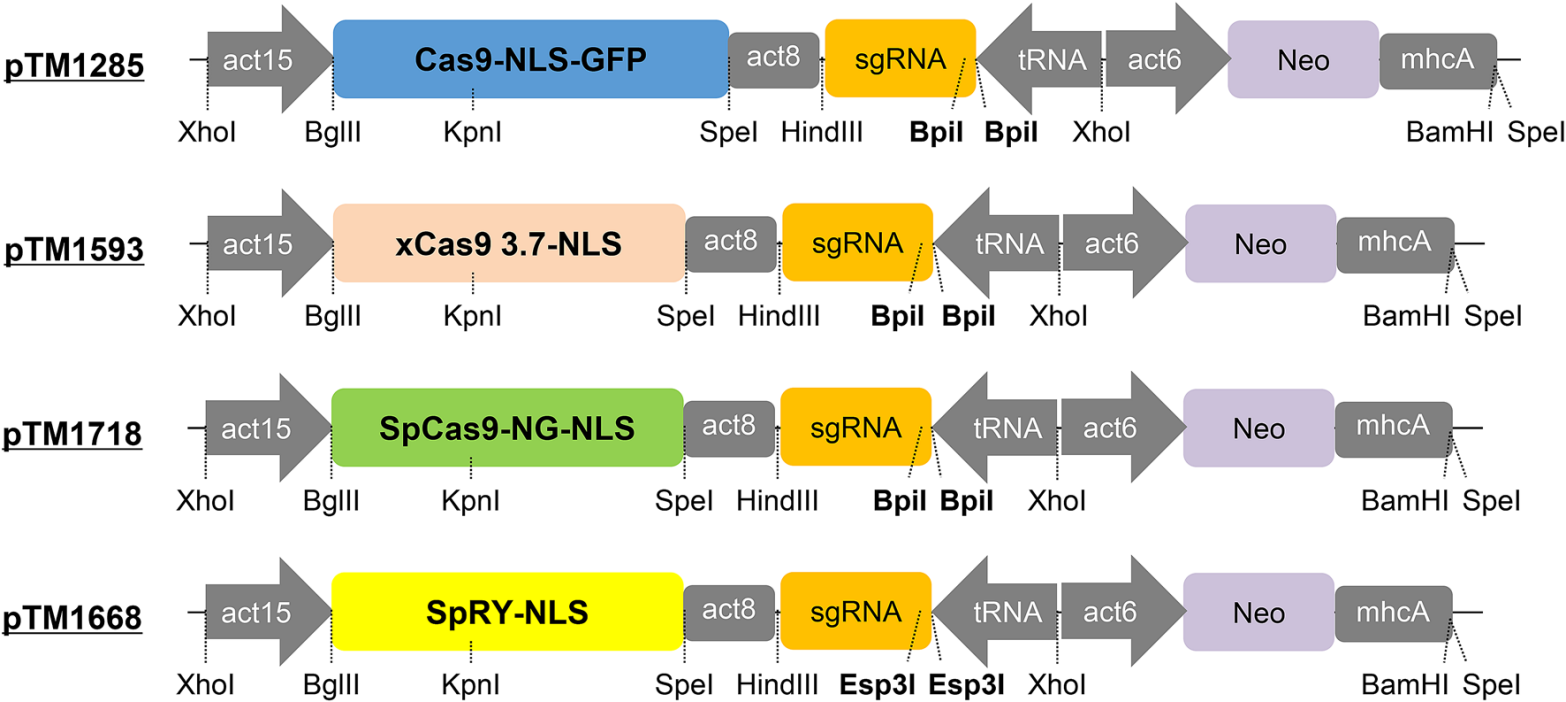
Diagrams of all-in-one Cas9 and sgRNA expression vectors used for gene manipulation in *D. discoideum*. act15, act15 promoter; act8, act8 terminator; tRNA, isoleucine tRNA; act6, act6 promoter; neo, neomycin resistance gene.

**Figure 2:**
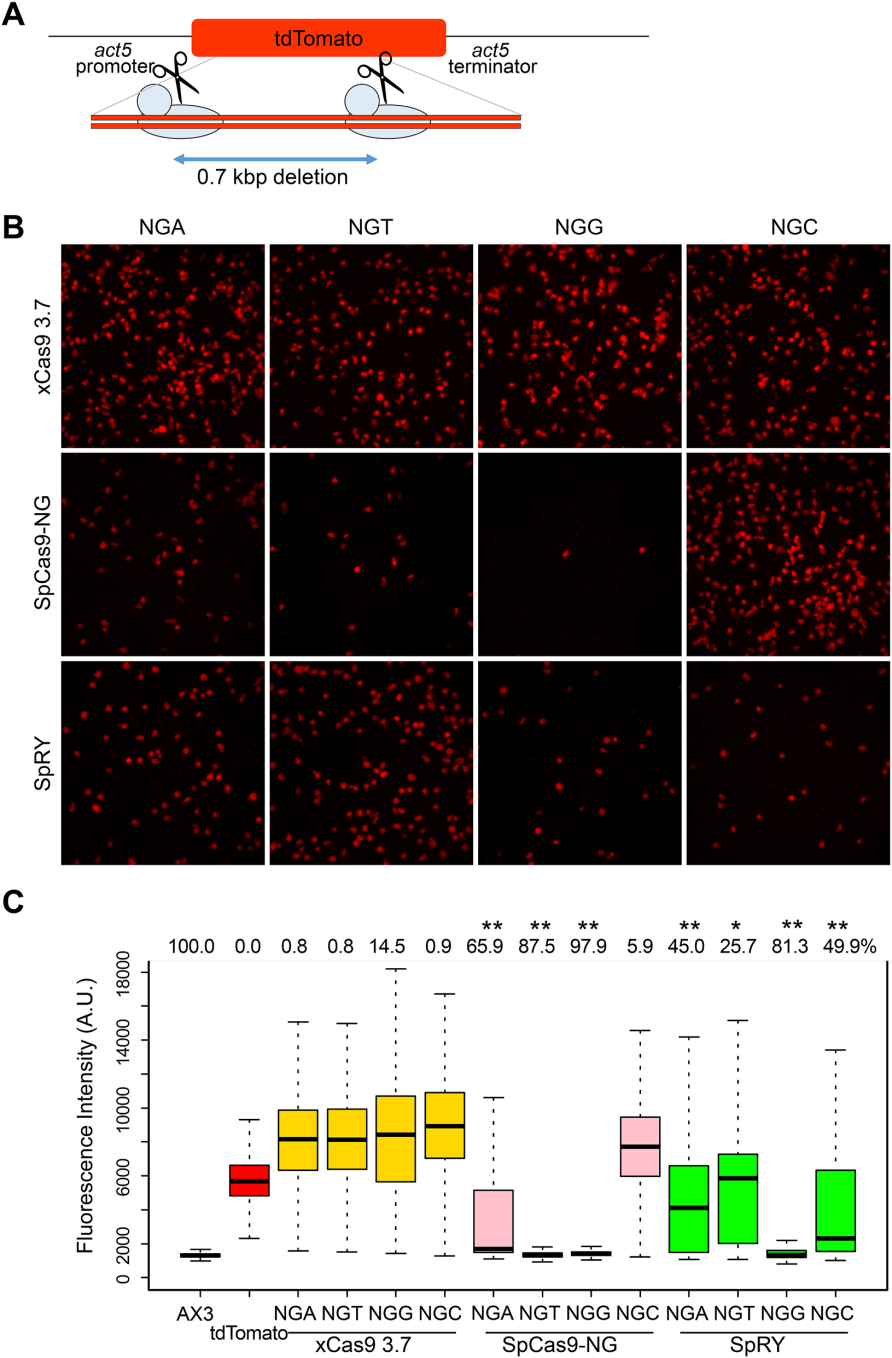
Gene mutagenesis by xCas9 3.7, SpCas9-NG, and SpRY in *D. discoideum*. (**A**) Schematic diagram of gene targeting in tdTomato knock-in cells. Because tdTomato contains tandem repeats, a 700 bp deletion can be generated with a single sgRNA. (**B**) Fluorescence observation of tdTomato in mutated cells. Four sgRNAs harbouring NGA, NGT, NGG or NGC PAMs were introduced into tdTomato-expressing cells along with the indicated Cas9 variants. Red fluorescence images of representative areas are shown. (**C**) Box plot represents loss of red fluorescence induced by Cas9 variants. AX3 indicates the auto-fluorescence level without tdTomato, and tdTomato indicates the expression level of the parental strain. Numbers above the graph show knockout efficiencies. *P = 0.064 and **P < 0.001; ANOVA followed by Tukey’s post hoc test. n = 3 biological replicates.

We constructed codon-optimised versions of Cas9 variants xCas9 3.7, SpCas9-NG, and SpRY, and then evaluated their editing efficiencies at various PAM sequences in *D. discoideum* (Fig. 1). In these experiments, we targeted the tdTomato gene, which was knocked in at the *act5* locus (Fig. 2A). Mutations led to a loss of fluorescence; thus, mutation efficiency could be measured by monitoring the percentage of non-fluorescent cells. Although tdTomato is not an endogenous gene in *D. discoideum*, the efficiencies we observed were almost identical to those of endogenous genes^11^. Four sgRNA sequences recognising different targets harbouring NGG, NGA, NGC or NGT PAMs were designed against the tdTomato gene (Table S1), and then inserted into all-in-one vectors containing various Cas9 variants (Tables S2 and S3). Classical SpCas9 edited DNA efficiently using a canonical NGG PAM, but only a few percent of cells lost red fluorescence when using non-canonical NGA, NGT, and NGC PAMs (Figs. S1 and S2). The initially generated all-in-one vector with classical SpCas9, pTM1285, contained a GFP sequence followed by Cas9. We also found that vectors without GFP, pTM1599 and pTM1644, could induce genome editing as efficiently as pTM1285 (Figs. S2 and S3).

In cells expressing a Cas9 variant and an sgRNA targeting the canonical NGG PAM, we detected loss of red fluorescence, as in cells expressing classical SpCas9. The mutation efficiencies of SpCas9-NG and SpRY were 97.8% and 81.3%, respectively, whereas that of xCas9 3.7 was only 14.5% (Fig. 2B,C). Loss of fluorescence due to xCas9 3.7-mediated editing at non-canonical PAMs was observed in less than 1% of cells, suggesting that xCas9 3.7 is not suitable for genome editing at NG PAM sequences in *D. discoideum*. On the other hand, SpCas9-NG and SpRY had efficiencies greater than 25%, except for SpCas9-NG at NGC, but none had higher efficiencies at these sequences than at the canonical NGG PAM. SpRY was more than 25% efficient with all NG PAMs, whereas SpCas9-NG exhibited higher or lower efficiencies at different PAMs.

**Figure 3:**
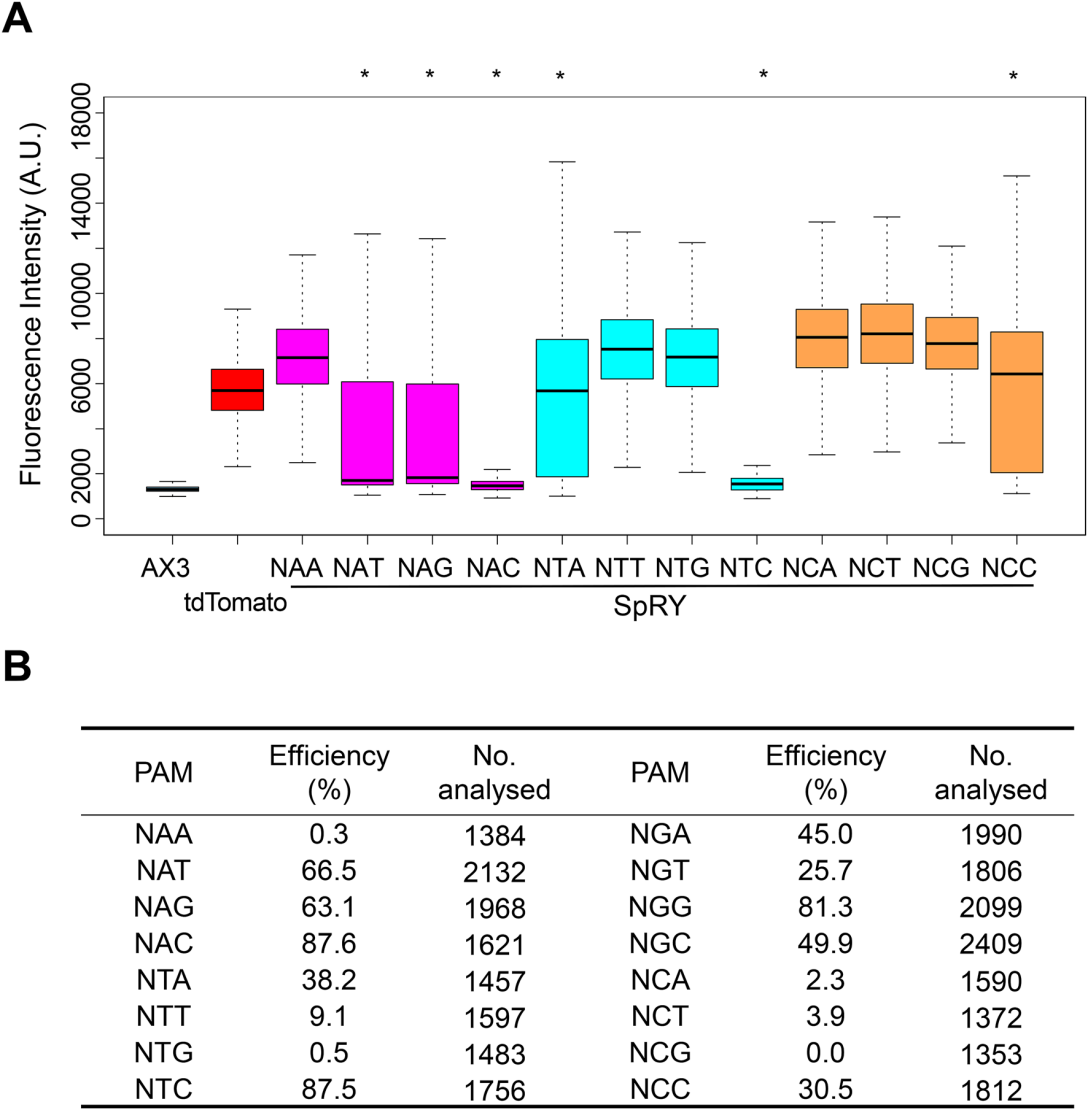
Targeting of non-canonical NHN PAMs in *D. discoideum* using SpRY. (**A**) Box plot represents fluorescence intensity with different sgRNAs in targets harbouring NHN PAMs. The target sites covered all twelve NHN PAM combinations. *P < 0.001; ANOVA followed by Tukey’s post hoc test. (**B**) Proportion of cells in which SpRY induced loss of fluorescence.

Because SpRY is a near-PAMless Cas9 variant (NRN > NYN)^29^, we next assessed SpRY editing activity while targeting non-canonical NHN PAM sites (where H is A, C, or T). For this purpose, we selected 12 targets harbouring NHN PAMs, all of which could target the tdTomato gene (Table S1). Half of the targets (6/12) exhibited highly efficient editing (25.7–87.6%) with SpRY, and two of them (NAC and NTC PAMs) were as or more efficient than the canonical NGG PAM (Fig. 3). Editing efficiency at NR (where R is A or G) was greater than 50% on average, whereas efficiencies at NT and NC were 33.8% and 9.2%, respectively, supporting the previous observation that SpRY is capable of efficiently targeting the majority of target sites with NRN PAMs^29^. Given that we observed highly efficient editing at almost all PAMs, including NGN PAMs, SpRY is likely to allow editing of the majority of genomic DNA in *D. discoideum*, with no restriction due to a requirement for NGG sequences.

### SpRY-mediated knock-in of tag-sequences

**Figure 4:**
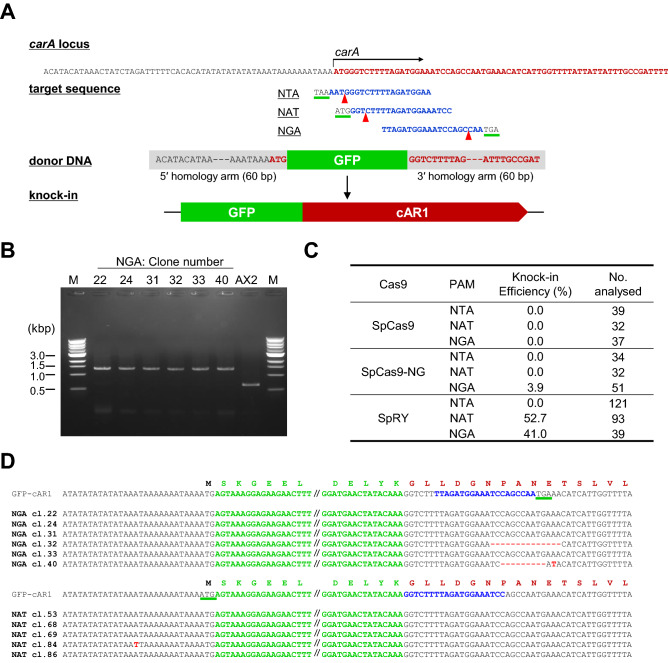
SpRY-mediated tag knock-in. (**A**) Schematic illustration of GFP knock-in at the cAR1 gene. Target sites in *carA* are in blue, and PAM sequences are underlined in green. Red arrowheads indicate sites of predicted cleavage by SpRY. Donor DNA was amplified by PCR using GFP primers flanked with homology arms (Table S4). (**B**) PCR amplification using primers flanking the knock-in site. AX2 presents the parental strain, and the rest of the lanes represent six individual mutants. An unprocessed image of gel shows that no other bands were present on the gel. (**C**) Summary of knock-in efficiencies with the indicated targets (NTA, NAT or NGA PAMs). (**D**) Sequencing results from the knock-in region. Sequences of GFP-cAR1 (top) and individual PCR positive mutants are shown. GFP sequences are in green, and target sequences are in blue. PAM sequences are underlined in green.

In cases of homologous recombination-mediated knock-in in *D. discoideum*, it is necessary to introduce a drug resistance cassette into the genome to allow isolation of transformants. It is challenging to insert a tag sequence inside a gene or immediately after the start codon without perturbation by the drug resistance cassette. It is possible to excise a loxP-flanked drug resistance cassette with a Cre protein, leaving the loxP sequence behind in the recombination region^31^. Overexpression of recombinant proteins with N-terminal tags or nucleotide substitutions is often used for functional analysis, but overproduction occasionally results in ectopic expression or dominant-negative effects. Because the nearly PAM-less Cas9 variant SpRY is functional in *D. discoideum*, and it is not necessary to introduce a drug resistance cassette into the genome, the tag sequence can be knocked in at any location, even in the middle of a gene. We selected the cAR1 gene as a target to investigate whether we could knock-in the GFP sequence immediately after the start codon. Design of target sequences within 30 bp of the start codon yielded 16 candidate sgRNAs with low off-target potential and high specificity for the desired region. Due to the low GC content within this region, these candidates did not include canonical NGG PAM targets. From the candidates, we selected target sequences with NTA, NAT, and NGA PAMs, and then generated all-in-one vectors containing SpCas9, SpCas9-NG, and SpRY, respectively (Table S3). To generate donor DNA containing the GFP sequence and the two homologous fragments (5′ and 3′ arms), we amplified the fragment by PCR using GFP primers flanked with 60 bp homology arms (Fig. 4A). Next, we simultaneously introduced the all-in-one vector and donor DNA into the AX2 strain to induce transient expression of the Cas9 and sgRNAs. We then isolated single clones and extracted the genomic DNA. Because the knocked-in clones yielded bands ~ 700 bp larger than the unedited clones, we could calculate the knock-in frequency by PCR (Fig. 4B). About 50% of the clones expressing SpRY with NAT and NGA PAMs contained knock-ins vs only a few percent of clones expressing SpCas9 and SpCas9-NG with NTA, NAT, and NGA PAMs (Fig. 4C). Sequence analysis confirmed that the correct knock-ins were present in more than 60% of the clones with NGA and NAT PAMs (Fig. 4D). Based on these results, we succeeded in generating a knock-in strain with only a tag sequence at an arbitrary part of the gene without introducing any extra sequences, such as drug resistance cassettes.

Homologous recombination for gene knock-out or knock-in in *D. discoideum* requires homology arms of 500 bp or longer^32^. In this study, we showed that CRISPR/Cas9 could efficiently generate a knock-in strain using homology arms of just 60 bp. However, the relationship between the length of the homology arms and knock-in efficiency remained unknown. Therefore, we generated donor DNA with 30 bp and 90 bp homology arms and compared the knock-in efficiencies. The 90 bp homology arms were roughly half as efficient as the 60 bp arms, whereas the knock-in efficiency of the 30 bp arms was significantly lower, indicating that 60 bp arms were sufficient (Fig. S4).

Overexpressed cAR1-GFP (a C-terminal GFP fusion of cAR1) is localised to the cell surface^33^. In GFP-cAR1 knock-in cells, localisation to the plasma membrane was very weak, even in cells in the aggregation stage (Fig. S5). This was because GFP was knocked in at the N-terminus, which is required for efficient translocation to the surface membrane. Formation of aggregation streams in submerged condition or on agar were not observed, and subsequent fruiting body formation was significantly delayed. However, unlike the cAR1 knockouts^34^, the mutants formed fruiting bodies within 2 days after starvation (Fig. S5).

### Accurate single-base substitution mediated by SpRY

**Figure 5:**
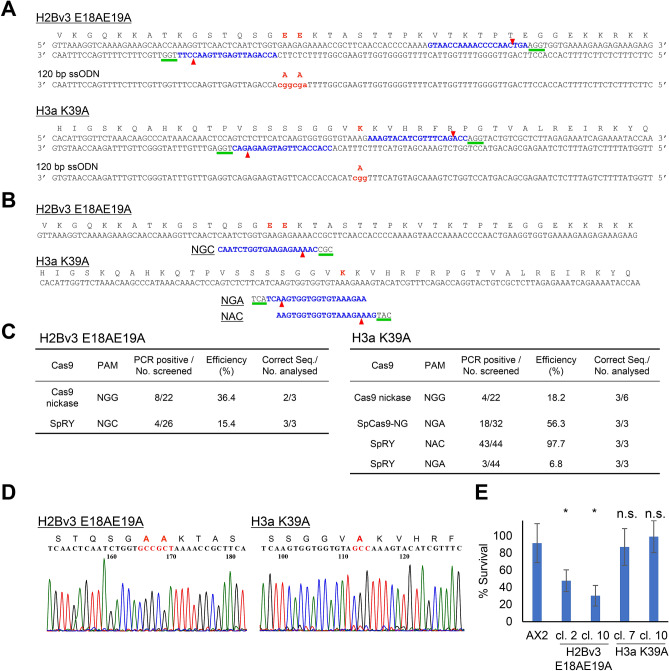
Precise nucleotide substitution mediated by double nicking and SpRY. (**A**) A pair of sgRNAs and ssODN for double-nicking. Point mutations are indicated by red letters. (**B**) Target sequences used for SpRY. Three targets with different PAMs are shown. (**C**) Summary of point mutation frequencies with the indicated CRISPR/Cas9 systems. (**D**) Sequencing chromatogram of each cell line. Mutated nucleotides and amino acids are shown in red. (**E**) Cell viability in response to phleomycin. Results are expressed as the percentage of point-mutated clones that survived. Error bars indicate S.D. of three biological replicates. *P < 0.001; n.s., not significant; Student’s t-test. n = 4 biological replicates.

For relatively short genes such as histones, accurate single-base substitution within the genome can be achieved by homologous recombination^35,36^. However, this approach is often challenging because it has low efficiency, generating only one positive out of hundreds of clones. To determine whether it would be possible to generate highly efficient single-base substitutions using the CRISPR/Cas9 system, we used Cas9 variants and Cas9 nickase. As targets, we selected H2Bv3 E18/E19 and H3a K39 (equivalent to K36 in mammals). Histone H2Bv3 E18/E19 is ADP-ribosylated in response to DNA double-strand breaks, and H3a K39 is methylated in association with active transcription at euchromatin^37,38^. We targeted the two histone sites with pairs of sgRNAs harbouring NGG PAM to generate double nicking mediated single-base substitution (Fig. 5A). All-in-one vectors expressing Cas9 nickase and sgRNAs were introduced into AX2 strains along with a single-stranded oligonucleotide (ssODN) containing a nucleotide substitution that would convert the corresponding amino-acid residue to alanine. Mutation detection PCR using primers with substituted nucleotides at the 3′ ends revealed that 18.2–36.4% of the independent clones yielded positive bands (Fig. 5C). In the case of these histones, we were able to design a pair of target sequences with canonical NGG PAMs around the nucleotide of interest. However, it is not always the case that two targets with NGG PAMs are located near a specific locus. Therefore, we used the Cas9 variants SpCas9-NG and SpRY to investigate whether a single-base substitution could be introduced into various targets harbouring non-canonical PAMs (Fig. 5A,B). PCR-positive base substitutions were present in 6.8–97.7% of the independent clones. In particular, base substitutions occurred in almost all (97.7%) of the clones expressing SpRY and sgRNA with the NAC PAM. To further evaluate the accuracy of single-base substitution, we analysed the nucleotide sequences; substitutions were present in 50–66% and 100% of PCR-positive clones expressing Cas9 nickase and Cas9 variants, respectively (Fig. 5C,D). E18 and E19 of H2Bv3 are ADP-ribosylated in response to DNA double-strand-breaks (DSBs)^37^. Hence, we investigated whether cells harbouring disruptions in histone modification sites were sensitive to the DSB-inducing reagent phleomycin. Cells harbouring H2Bv3 E18A E19A were more sensitive than AX2 to phleomycin, whereas cells harbouring a point mutation in H3a K39, a mark of active transcription, were insensitive (Fig. 5E).

## Discussion

The ability to perform gene modification in regions that are not accessible by conventional SpCas9 expands the toolbox for editing the AT-rich genome of *D. discoideum*. We successfully used the Cas9 variants xCas9 3.7, SpCas9-NG, and SpRY to perform genome editing at various PAM sites. In particular, with SpRY, half of the targets harbouring NHN PAMs were editable with high efficiency. As previously shown for CRISPR/Cas9-mediated genome editing in *D. discoideum*, it is relatively easy to design target sequences within a gene of interest that contains an NGG PAM^11,12,39^. In fact, we can design target sequences with high specificity for all 283 of the known kinase genes. On the other hand, the PAM requirement is a severe barrier for applications other than gene knock-out, such as nucleotide substitution, targeting of a narrow genetic region, and tiling of regulatory elements^40–42^. For example, if we wished to design target sequences with NGG PAMs that cleave 25 bp before and after the start codon, we would not be able to target 251/283 (88.7%) of the kinase genes. Thus, SpCas9-NG and SpRY represent useful alternatives to SpCas9, and could be used to generate a variety of mutations that require precise DNA breaks at positions of interest not accessible by NGG PAMs.

The editing efficiencies of the three Cas9 variants were similar to those reported in other organisms, and the low editing efficiency of xCas9 3.7 at NG PAMs was consistent with results obtained in plants^24,29,30,43^. However, stable transgenic rice lines revealed that xCas9 3.7 functioned efficiently, with mutation rates of approximately 80% at NG PAM sites^25^. It is unclear whether the differences in editing efficiencies are due to stable versus transient expression or differences in other components. Recently, new SpCas9 variants that recognise NRNN PAMs have been engineered via the continuous evolution strategies used to generate xCas9 3.7^44^. Because xCas9 3.7 has high fidelity^45^, future improvement is likely to make xCas9 a more practical choice for targeting non-NGG sites. Although SpCas9-NG and SpRY are presumed to generate more off-target effects due to the relaxed PAM requirements, SpCas9-NG had similar or even higher editing specificity in mammalian cells and rice^24,26^. SpRY was more prone to off-target editing than SpCas9, but these off-target effects could be eliminated by combining high-fidelity variants^29,46,47^. In *D. discoideum*, CRISPR/Cas9-mediated genome editing is achieved through transient expression of the all-in-one vector. Consequently, off-target effects are minimised, and elimination of the vectors can be confirmed by re-exposure to G418 selection. However, unintended editing remains a significant problem, and future studies should seek to further minimise this effect.

Importantly, we only tested a subset of PAMs. Because differences in 4 and 5 nt PAM sequences affect the editing efficiency^45^, more targets must be tested to accurately determine efficiencies in *D. discoideum*. The lack of GFP knock-in at cAR1 with the NTA PAM, which had an efficiency of ~ 30% in tdTomato editing using SpRY, could be due to the fourth nucleotide of the PAM sequence. Although fine-tuning of PAM sequences requires further analysis, one effective method for generating mutants would be to design multiple targets for genome editing.

Previously, several 5′- or 3′-terminal knock-in mutants were successfully generated via homologous recombination in *D. discoideum*^48–50^. To date, however, no study has reported knock-in of GFP alone at the 5′-end of a gene. Even when GFP has been integrated at the 5′-end of a gene, the drug resistance cassette and *act6* promoter for induction of target gene expression were introduced as extra sequences, and induction of expression could not be achieved under the control of the endogenous promoter^48^. Using the CRISPR-based knock-in method described here, it is possible to introduce the tag sequence anywhere in the gene of interest, allowing for analysis of tagged proteins expressed at approximately the same levels as the endogenous target proteins. This would make it possible to introduce tags into a gene that cannot be analysed with overexpression vectors. In addition, it would facilitate more accurate measurements of the number of intracellular molecules in live-imaging studies, such as Fluorescence Resonance Energy Transfer (FRET) experiments in G proteins^51^ and GFP-tagging experiments to monitor protein expression levels^52^. For nucleotide substitution, base editors have frequently been used in various organisms as an alternative tool to HDR-mediated precise nucleotide substitution^53–56^. Although we have not used cytidine base editor (CBE) and adenine base editor (ABE) in *D. discoideum*, in this study we achieved HDR-mediated nucleotide substitution with efficiency of up to 97.7% using SpRY. Therefore, generation of amino-acid substitutions with SpRY could replace the traditional approach in which a gene with a point mutation is overexpressed in *D. discoideum*.

Our results show that the Cas9 variants used in this study substantially expand the utility of knock-in at a precise position within a target gene in *D. discoideum*. The versatile toolboxes developed here significantly expand the potential genome editing, making the majority of the *D. discoideum* editable.

## Methods

### Plasmid constructs

Coding sequences of xCas9 3.7, SpCas9-NG and SpRY followed by an NLS were codon-optimised for *D. discoideum* using the Codon Optimization Tool from Integrated DNA Technologies (IDT). The resultant 4.2 kb DNA sequences were divided into five fragments and synthesised as gBlocks (IDT) with 15 nt flanking sequences allowing In-Fusion HD cloning (Takara Bio). The first two and second three fragments were cloned into pBlueScriptII, and the sequences were confirmed by Sanger sequencing. The two fragments were joined using the KpnI site within Cas9 to obtain a full-length Cas9 fragment. The BglII- and SpeI-flanked Cas9 variants encoding xCas9 3.7 and SpCas9-NG were cloned into pTM1285 by replacing the Cas9-NLS-GFP sequence. The SpRY coding sequence was cloned into pTM1416 by replacing Cas9-NLS-GFP. pTM1416 was constructed by converting the two BpiI sites of the tRNA–sgRNA cassette in pTM1285 to two Esp3I sites by inserting a pair of annealed oligonucleotides, 5′-AGCAGGAGACGGGCGTCTCG-3′ and 5′-AAACCGAGACGCCCGTCTCC-3′. We also constructed the SpCas9-NG vector, which contained an tRNA–sgRNA cassette with two Esp3I sites (pTM1719), by replacing the two BpiI sites in pTM1718. To construct SpCas9-NLS vectors without GFP, we eliminated the GFP sequence in pTM1285 and pTM1416 by digestion with ClaI and SpeI, followed by blunting and self-ligation to yield pTM1599 and pTM1644, respectively (Table S2). Predicted nucleotide sequences for the all-in-one vectors are shown in Fig. S6. These plasmids will be made available to all researchers via NBRP Nenkin and other stock centres.

### Design and cloning of sgRNAs

sgRNAs for SpCas9 were designed by identifying the NGG PAM sequence using CRISPR RGEN^57^. The freely available stand-alone Python scripts Cas-designer, Cas-OFFinder-bulge, and Cas-OFFinder Binaery ver. 2.4 were used to design 15 targeting sequences (NNN PAM with the exception of NGG PAM)^58^. Genome sequence (dicty_2.7) was downloaded via the Ensembl genome browser and converted to a 2-bit file that was smaller than the corresponding FASTA format. Sequences for target genes were saved in FASTA format. Output data were filtered as follows: GC content 30–75%, out-of-frame scores > 66, and mismatch number “1,0,0”. In the case of duplicated genes such as tdTomato or *carA*, mismatch numbers “2,0,0” was used. The 20 nt targeting sequences without PAMs were synthesised as pairs of oligonucleotides with overhang sequences AGCA (forward) and AAAC (reverse) (Table S1). After mixing the two oligonucleotides (10 µM each) in annealing buffer (40 mM Tris–HCl pH 8.0, 20 mM MgCl_2_, 50 mM NaCl), the mixture was heated at 95 °C for 5 min and cooled to 25 °C (1 °C/min). The annealed oligonucleotides (1.0 µl) were then ligated into an all-in-one vector (25 ng) via a Golden Gate digestion/ligation reaction using 70 U T4 DNA ligase and 1.5 U BpiI (Thermo) or Esp3I (NEB) in 4.0 µl reactions. The reactions were placed in a thermal cycler and subjected to eight cycles of 37 °C for 5 min and 16 °C for 17 min. After the Golden Gate reaction, additional BpiI- or Esp3I-digestion was performed at 37 °C for 60 min to remove all unligated all-in-one vectors. Transformed colonies were analysed for insertion of target sequence via colony PCR using forward target oligonucleotide and tracr-Rv (Tables S1 and S4). PCR primers were designed to amplify target sequence and tracrRNA within the sgRNAs such that the correct PCR band was ~ 120 bp. When further validation of the insert was necessary, sequencing analysis was performed using NeoUp (Table S4). For Cas9 nickase, a pair of target sequences with PAMs facing outward were selected and synthesised with overhangs appropriate for one-step Golden Gate as follows (Table S5). A pair of annealed oligonucleotides (0.26 µl each) prepared as described above, was ligated into pTM1544 (40 ng) via the Golden Gate reaction using 140 U T4 DNA ligase and 3.0 U BpiI (Thermo) in 8.0 µl reactions. Correct assembly of dual-sgRNA was confirmed via colony PCR using GFPdown and Reverse (+ direction) primers for the first target, and NeoUp2 and Forward (− direction) primers for the second target (Tables S4 and S5).

### Cell culture, transformation, and identification of transformants

Axenic strains AX2 and tdTomato knock-in cells in the AX3 background were cultured at 22 °C in HL5 medium or on SM agar plates with *Klebsiella pneumoniae* (KpGe)^59^. To obtain cells transiently expressing Cas9 and sgRNAs, 10 µg all-in-one vector was transformed into cells using H50 buffer^11,12^. For knock-in or point mutagenesis, 2.4 µg donor DNA or 2.5 µl of 10 µM ssODN were added, respectively. After electroporation, the cells were cultured in HL5 for 7–24 h and then maintained for another 1–2 days in HL5 containing 10 µg/ml G418. As the cells became rounded, they were recovered in HL5 without G418. To isolate single clones, the cells were plated on SM agar plates and incubated for 4 days until plaques formed. Genomic DNA from single clones was isolated in lysis buffer (1× PCR buffer, 0.5% NP40 and 50 µg/ml of Proteinase K), and the suspension was incubated at 56 °C for 45 min followed by 95 °C for 10 min. Cell lysate was used as a template for PCR to detect mutations mediated by CRISPR/Cas9.

### Calculation of targeting efficiency

Targeting efficiency mediated by Cas9 variants was calculated by monitoring the decrease in tdTomato fluorescence within a single cell. Equal numbers of cells (1.8 × 10^5^ cells/cm^2^) were plated on Nunc Lab-Tek II two-well chambered coverglass. To visualise individual cells, their nuclear were stained with 10 µg/ml DAPI for 25 min. Blue and red fluorescence images were acquired on an Olympus IX71 inverted fluorescence microscope with a 0.75 NA 40× objective and Orca-Flash4.0 V2 Digital CMOS camera (Hamamatsu). We measured the intensity of tdTomato by averaging the intensity within individual cells using Volocity (Perkin Elmer). The efficiency was determined by pooling the intensity data of individual cells obtained from at least three independent transformations per vector. Cells that exhibited lower red fluorescence than tdTomato knock-in cells were defined as knockouts. Targeting efficiencies of knock-ins were calculated by PCR using primers flanking the target sites; efficiencies of point mutations were calculated using primers with substituted nucleotides at the 3′ ends (Table S4).

### DNA damage assay

Growing cells were plated on 24-well culture dishes at 2.5 × 10^5^ cells/cm^2^ and exposed to 10 µg/ml phleomycin (Cayman Chemical) or mock-treated for 30 min. Cell viability was determined by plating 100 cells on three SM agar plates and counting the number of plaques after 4–6 days.

### Statistical analysis

We used a one-way ANOVA followed by Tukey’s post hoc test for the comparison of knock-out frequencies between PAM groups. The mean values of the survival rates of the wild-type and mutants in the DNA damage assay were considered to be equal using Student’s t-test under the null hypothesis.

## Supplementary information

Supplementary Informations.
