# Supplementary material for: Knock-in and precise nucleotide substitution using near-PAMless engineered Cas9 variants in Dictyostelium discoideum
Source: Sci Rep. 2021 May 27;11:11163. doi: 10.1038/s41598-021-89546-0 (PMC8159936; doi:10.1038/s41598-021-89546-0)
Supplement: Supplementary file 1 — Supplementary Informations. [file 41598_2021_89546_MOESM1_ESM.pdf]

Knock-in and precise nucleotide substitution using near-PAMless engineered Cas9 variants in *Dictyostelium discoideum*

Yuu Asano#, Kensuke Yamashita#, Aoi Hasegawa, Takanori Ogasawara, Hoshie Iriki, Tetsuya Muramoto*

Department of Biology, Faculty of Science, Toho University, 2-2-1 Miyama, Funabashi, Chiba 274-8510, Japan.

* Corresponding author:
tetsuya.muramoto@sci.toho-u.ac.jp
Department of Biology, Faculty of Science, Toho University, 2-2-1 Miyama, Funabashi, Chiba 274-8510, Japan.
# Authors contribute equally

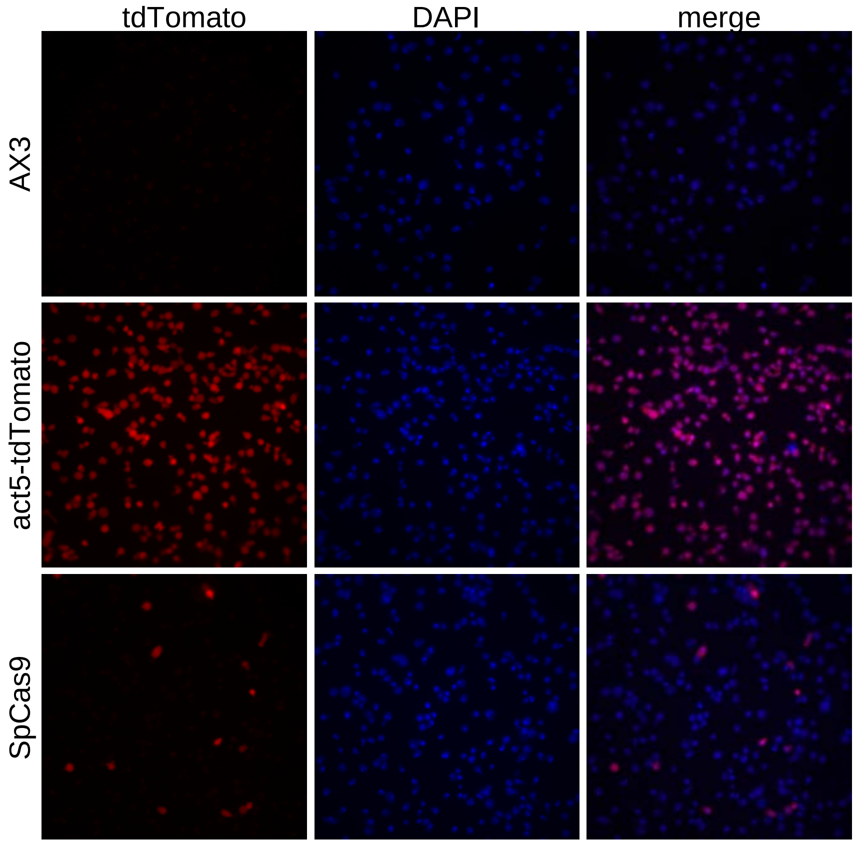

Supplementary Figure S1. SpCas9-mediated targeting was monitored via fluorescence images of tdTomato. The same number of cells were plated in an imaging chamber and stained with DAPI to visualise individual nuclei.

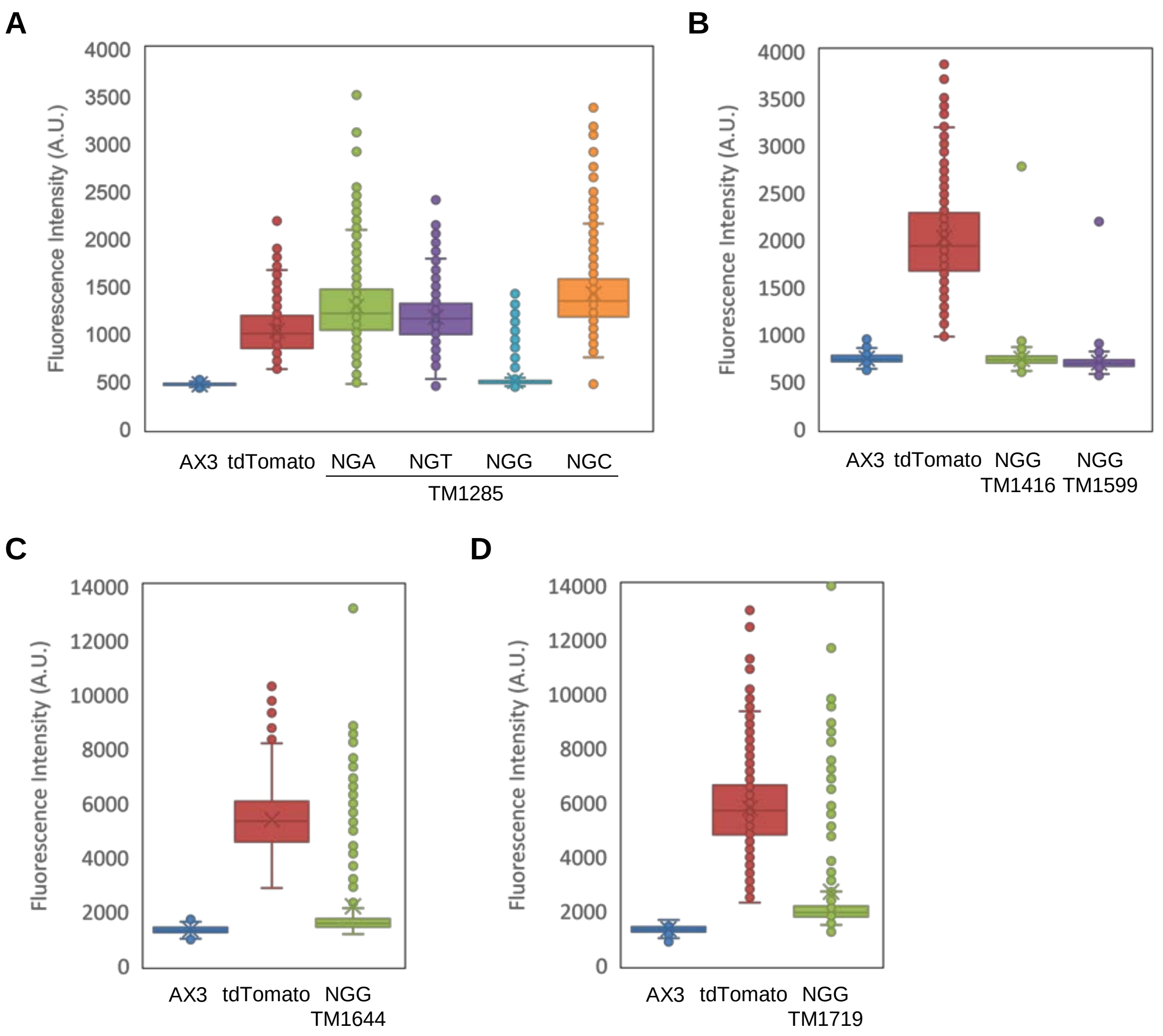

E

Vector	pTM1285				pTM1416	pTM1599	pTM1644	pTM1719
PAM	NGA	NGT	NGG	NGC	NGG	NGG	NGG	NGG
Efficiency (%)	2.0%	3.6%	97.6%	0.7%	99.6%	99.8%	86.3%	79.8%
No. analysed	1091	468	701	837	963	572	461	332

Supplementary Figure S2. Gene mutagenesis by various Cas9 constructs in *D. discoideum*.
(A) Loss of fluorescence induced by SpCas9 (pTM1285) and various NG PAMs. (B) Genome editing via all-in-one vectors with SpCas9, pTM1416 and pTM1599. (C) Genome editing via all-in-one vector pTM1644. (D) Genome editing mediated by the SpCas9-NG expression vector pTM1719. (E) Summary of genome editing efficiencies with the indicated CRISPR/Cas9 vectors and PAMs.

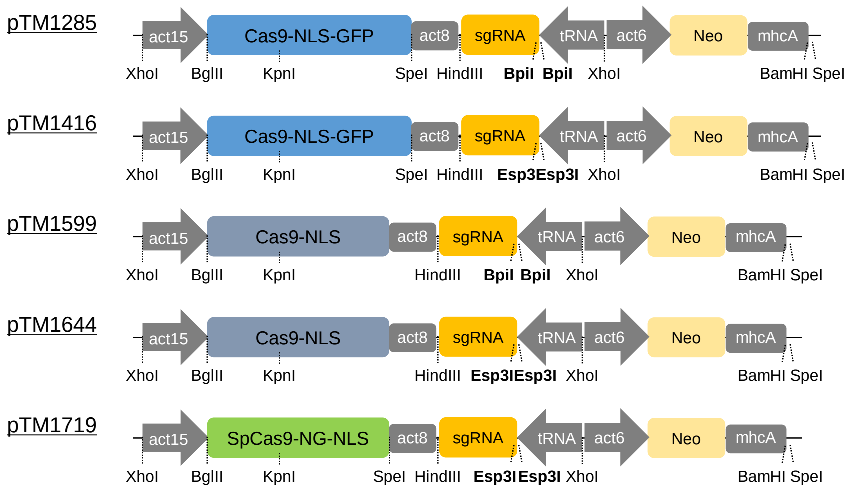

Supplementary Figure S3. Diagrams of various Cas9 and sgRNA expression vectors. act15, act15 promoter; act8, act8 terminator; tRNA, isoleucine tRNA; act6, act6 promoter; neo, neomycin resistance gene.

A

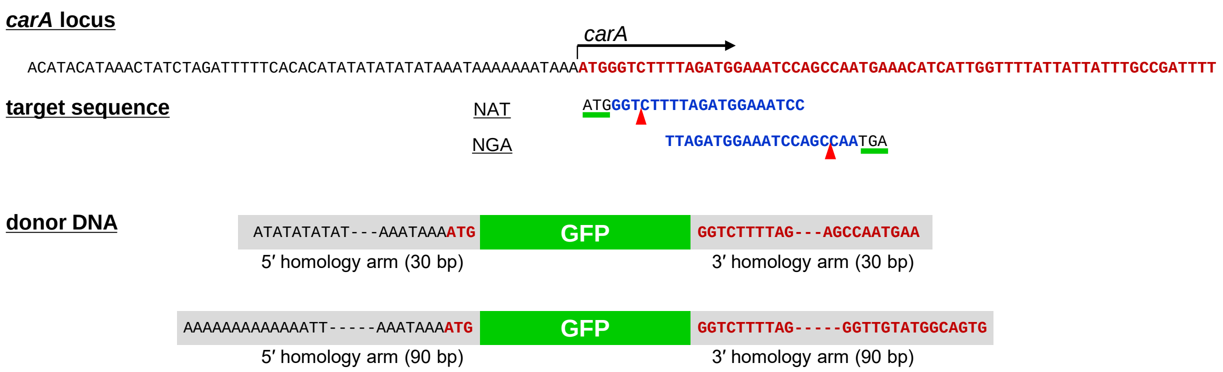

B

Cas9	PAM	Homology arm	PCR positive / No. screened	Efficiency (%)	Correct Seq./ No. analysed
SpRY	NAT	30 bp	9/38	23.7	3/5
SpRY	NAT	60 bp	49/93	52.7	4/5
SpRY	NAT	90 bp	11/38	28.9	5/5
SpRY	NGA	30 bp	2/48	4.2	0/2
SpRY	NGA	60 bp	16/39	41.0	4/6
SpRY	NGA	90 bp	11/47	23.4	5/5

Supplementary Figure S4. Knock-in frequencies depending on the length of homology arms in donor DNA. (A) Diagrams of GFP knock-in at the cAR1 gene with the indicated donor DNAs. (B) Summary of knock-in efficiencies with homology arms of various lengths.

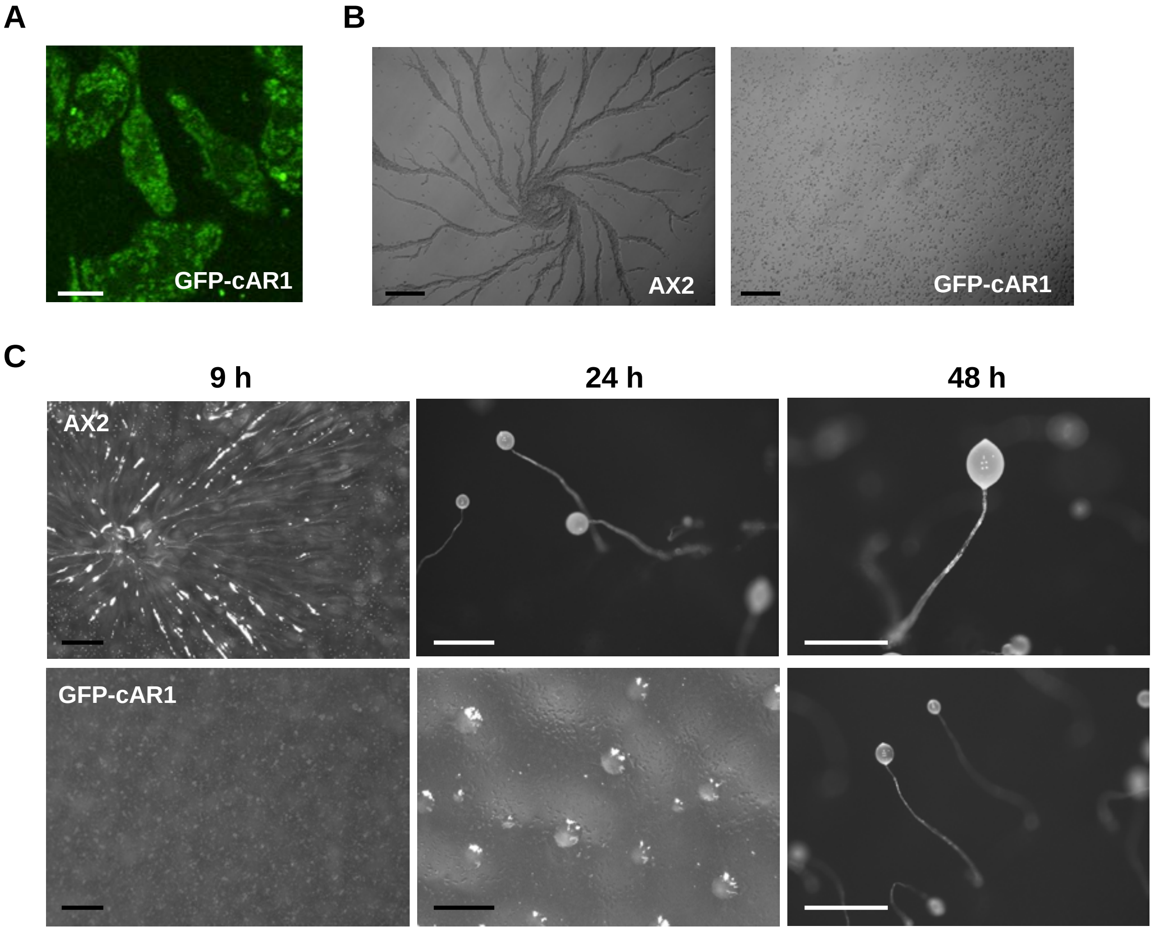

Supplementary Figure S5. Phenotype of GFP-cAR1 knock-in cells.
(A) Localisation of GFP-cAR1 during the aggregation stage. Bar is 10 µm in length. (B) Aggregation under submerged conditions. Cells were washed and incubated for 8 h in a 24-well plate with DB buffer at a density of 2.0×10^5 cells/cm^2. Bars represent 0.2 mm. (C) Developmental phenotype of GFP-cAR1 knock-in cells. Cells were plated on agar, and images were acquired at the indicated times. Bars represent 0.5 mm.

```
>pTM1593(xCas9 3.7-NLS)
CTAAATTGTAAGCGTTAATATTTTGTTAAAATTCGCGTTAAATTTTTGTTAAATCAGCTCATTTTTTAACCAATAGGCCGAAATCGGCAAAATCCCTTATAAATCAAAAGAATAGACCGAGATAGGG
TTGAGTGTTGTTCCAGTTTGGAACAAGAGTCCACTATTAAAGAACGTGGACTCCAACGTCAAAGGGCGAAAAACCGTCTATCAGGGCGATGGCCCACTACGTGAACCATCACCCTAATCAAGTTTTT
TGGGGTCGAGGTGCCGTAAAGCACTAAATCGGAACCCTAAAGGGAGCCCCCGATTTAGAGCTTGACGGGGAAAGCCGGCGAACGTGGCGAGAAAGGAAGGGAAGAAAGCGAAAGGAGCGGGCGCTAG
GGCGCTGGCAAGTGTAGCGGTCACGCTGCGCGTAACCACCACACCCGCCGCGCTTAATGCGCCGCTACAGGGCGCGTCCCATTCGCCATTCAGGCTGCGCAACTGTTGGGAAGGGCGATCGGTGCGG
GCCTCTTCGCTATTACGCCAGCTGGCGAAAGGGGGATGTGCTGCAAGGCGATTAAGTTGGGTAACGCCAGGGTTTTCCCAGTCACGACGTTGTAAAACGACGGCCAGTGAGCGCGCGTAATACGACT
CACTATAGGGCGAATTGGGTACCGGGCCCCCCCTCGAGACTAGATAAAAAAAATTTTTATTTATTTTTATTTATTTTGAATTAAATAGATTACAAATTAATTAATCCCATCAAATCTTTAAAAAAAA
AAATGGTTTAAAAAACTTGGGTTGGTTAATTATTATTTGAAAATTTTAAAACCCAAATTAAAAAAAAAAAAATGGGATTCAAAAATTTTTTTTTTTTTTTTTTTTTTTTTTTTTTTTTTTTCAGATTGCA
TAAAAAGATTTTTTTTTTTTTTTTTTTCTTATTTCTTAAAACAAATAAATTAAATTAAATAAAAAATAAAAATCAGATCTATACAACAATAAAATGGATAAAAAATATTCAATTGGTTTAGATATTG
GTACAAATAGTGTTGGTTGGGCAGTTATTACAGATGAATATAAAGTTCCATCAAAAAAATTTAAAGTTTTAGGTAATACAGACCGGCATTCCATCAAGAAAAATCTGATCGGAGCTCTCCTCTTTGA
TTCAGGGGAGACCGCTGAAGCAACCCGCCTCAAGCGGACTGCTAGACGGCGGTACACCAGGAGGAAGAACCGGATTTGTTACCTTCAAGAGATATTCTCCAACGAAATGGCAAAGGTCGACGACAGC
TTCTTCCATAGGCTGGAAGAATCATTCCTCGTGGAAGAGGATAAGAAGCATGAACGGCATCCCATCTTCGGTAATATCGTCGACGAGGTGGCCTATCACGAGAAATACCCAACCATCTACCATCTTC
GCAAAAAGCTGGTGGACTCAACCGACAAGGCAGACCTCCGGCTTATCTACCTGGCCCTGGCCCACATGATCAAGTTCAGAGGCCACTTCCTGATCGAGGGCGACCTCAATCCTGACAATAGCGATGT
GGATAAACTGTTCATCCAGCTGGTGCAGACTTACAACCAGCTCTTTGAAGAGAACCCCATCAATGCAAGCGGAGTCGATGCCAAGGCCATTCTGTCAGCCCGGCTGTCAAAGAGCCGCAGACTTGAG
AATCTTATCGCTCAGCTGCCGGGTGAAAAGAAAAATGGACTGTTCGGGAACCTGATTGCTCTTTCACTTGGGCTGACTCCCAATTTCAAGTCTAATTTCGACCTGGCAGAGGATACTAAATTGCAAC
TTAGTAAAGATACATACGATGATGATCTTGATAATTTGTTGGCACAAATAGGAGATCAATATGCTGATTTGTTTTTAGCTGCCAAAAATTTGAGTGATGCCATACTTTTGTCAGATATTTTGAGAGT
TAATACCGAAATTACCAAAGCACCACTTTCAGCTAGTATGATTAAGTTATATGATGAACACCACCAAGATTTAACTTTATTGAAAGCATTGGTTCGTCAACAATTGCCTGAAAAGTATAAGGAAATC
TTCTTTGATCAATCAAAAAATGGATACGCTGGTTACATAGATGGTGGTGCCTCTCAAGAGGAGTTTTATAAATTCATCAAGCCAATTTTGGAGAAGATGGATGGTACTGAGGAACTTTTAGTTAAAC
TTAATAGAGAAGATTTATTGCGTAAGCAACGTACATTTGATAATGGAATAATTCCTCATCAAATACACTTGGGAGAGTTACACGCTATCCTTCGTCGTCAAGAAGATTTCTACCCTTTCCTTAAAGA
TAATCGTGAGAAGATAGAGAAAATCCTTACTTTCCGTATTCCTTATTATGTTGGTCCTTTAGCAAGAGGTAATTCTCGTTTTGCATGGATGACCAGAAAGTCTGAGGAAACAATCACTCCATGGAAT
TTTGAAAAGGTAGTTGATAAGGGAGCTAGTGCACAAAGTTTCATCGAGCGTATGACTAATTTTGATAAGAATCTTCCAAATGAGAAGGTATTGCCTAAGCACTCACTTCTTTACGAGTATTTCACCG
TATATAATGAGTTGACCAAGGTTAAGTACGTAACTGAGGGAATGCGTAAGCCTGCTTTTCTTTCTGGAGATCAAAAGAAAGCCATTGTTGATTTGTTATTCAAGACTAATAGAAAGGTAACAGTAAA
GCAACTTAAAGAGGATTACTTTAAGAAAATAGAGTGTTTCGATAGTGTTGAGATAAGTGGTGTAGAGGATAGATTTAATGCATCATTGGGTACCTACCATGATCTGCTTAAGATCATCAAGGACAAG
GATTTTCTGGACAATGAGGAGAACGAGGACATCCTTGAGGACATTGTCCTGACTCTCACTCTGTTCGAGGACCGGGAAATGATCGAGGAGAGGCTTAAGACCTACGCCCATCTGTTCGACGATAAAG
TGATGAAGCAACTTAAACGGAGAAGATATACCGGATGGGGACGCCTTAGCCGCAAACTCATCAACGGAATCCGGGACAAACAGAGCGGAAAGACCATTCTTGATTTCCTTAAGAGCGACGGATTCGC
TAATCGCAACTTCATACAACTTATCCATGATGATTCCCTGACCTTTAAGGAGGACATCCAGAAGGCCCAAGTGTCTGGACAAGGTGACTCACTGCACGAGCATATCGCAAATCTGGCTGGTTCACCC
GCTATTAAGAAGGGTATTCTCCAGACCGTGAAAGTCGTGGACGAGCTGGTCAAGGTGATGGGTCGCCATAAACCAGAGAACATTGTCATCGAGATGGCCAGGGAAAACCAGACTACCCAGAAGGGAC
AGAAGAACAGCAGGGAGCGGATGAAAAGAATTGAGGAAGGGATTAAGGAGCTCGGGTCACAGATCCTTAAAGAGCACCCGGTGGAAAACACCCAGCTTCAGAATGAGAAGCTCTATCTGTACTACCT
TCAAAATGGACGCGATATGTATGTGGACCAAGAGCTTGATATCAACAGGCTCTCAGACTACGACGTGGACCATATCGTCCCTCAGAGCTTCCTCAAAGACGACTCAATTGACAATAAGGTGCTGACT
CGCTCAGACAAGAACCGGGGAAAGTCAGATAACGTGCCCTCAGAGGAAGTCGTGAAAAAGATGAAGAACTATTGGCGCCAGCTTCTGAACGCAAAGCTGATCACTCAGCGGAAGTTCGACAATCTCA
CTAAGGCTGAGAGGGGCGGACTGAGCGAACTGGACAAAGCAGGATTCATTAAACGGCAACTTGTGGAGACTCGGCAGATTACTAAACATGTCGCCCAAATCCTTGACTCACGCATGAATACCAAGTA
CGACGAAAACGACAAACTTATCCGCGAGGTGAAGGTGATTACCCTGAAGTCCAAGCTGGTCAGCGATTTCAGAAAGGACTTTCAATTCTACAAAGTGCGGGAGATCAATAACTATCATCATGCTCAT
GACGCATATCTGAATGCCGTGGTGGGAACCGCCCTGATCAAGAAGTACCCAAAGCTGGAAAGCGAGTTCGTGTACGGAGACTACAAGGTCTACGACGTGCGCAAGATGATTGCCAAATCTGAGCAGG
AGATCGGAAAGGCCACCGCAAAGTACTTCTTCTACAGCAACATCATGAATTTCTTCAAGACCGAAATCACCCTTGCAAACGGTGAGATCCGGAAGAGGCCGCTCATCGAGACTAATGGGGAGACTGG
CGAAATCGTGTGGGACAAGGGCAGAGATTTCGCTACCGTGCGCAAAGTGCTTTCTATGCCTCAAGTGAACATCGTGAAGAAAACCGAGGTGCAAACCGGAGGCTTTTCTAAGGAATCAATCCTCCCC
AAGCGCAACTCCGACAAGCTCATTGCAAGGAAGAAGGATTGGGACCCTAAGAAGTACGGCGGATTCGATTCACCAACTGTGGCTTATTCTGTCCTGGTCGTGGCTAAGGTGGAAAAAGGAAAGTCTA
AGAAGCTCAAGAGCGTGAAGGAACTGCTGGGTATCACCATTATGGAGCGCAGCTCCTTCGAGAAGAACCCAATTGACTTTCTCGAAGCCAAAGGTTACAAGGAAGTCAAGAAGGACCTTATCATCAA
GCTCCCAAAGTATAGCCTGTTCGAACTGGAGAATGGGCGGAAGCGGATGCTCGCCTCCGCTGGCGTACTTCAGAAGGGTAATGAGCTGGCTCTCCCCTCCAAGTACGTGAATTTCCTCTACCTTGCA
AGCCATTACGAGAAGCTGAAGGGGAGCCCCGAGGACAACGAGCAAAAGCAACTGTTTGTGGAGCAGCATAAGCATTATCTGGACGAGATCATTGAGCAGATTTCCGAGTTTTCTAAACGCGTCATTC
TCGCTGATGCCAACCTCGATAAAGTCCTTAGCGCATACAATAAGCACAGAGACAAACCAATTCGGGAGCAGGCTGAGAATATCATCCACCTGTTCACCCTCACCAATCTTGGTGCCCCTGCCGCATT
CAAGTACTTCGACACCACCATCGACCGGAAACGCTATACCTCCACCAAAGAAGTGCTGGACGCCACCCTCATCCACCAGAGCATCACCGGACTTTACGAAACTCGGATTGACCTCTCACAGCTCGGA
GGGGATGAGGGAGCTCCCAAGAAAAAGCGCAAGGTAGGTAGTTCCTAAACTAGTTAAATAAATAAATTATTTAATAAATAATAAAAAAACAAATTGTTGTAATAATCTAATATTTTCTTTTTTTTTT
AATTTTTTTTTTTTAAATCTTAATAATTATTAAGTTATTTTAATTTTTTTTTTTTTTTTTTTTTTTTTTTTTTTTTTTTTTTCTATCAAAAAAATCAAATATATTTAAAAAATTTATTATTTACAGATACA
TTTTGAATGGTGAAGATAAATATATGCATTAGATGTAAAACAGCCAAAGAGTATGAAAATCAAAAAGATAAAGCTTAAAAAAAGCACCGACTCGGTGCCACTTTTTCAAGTTGATAACGGACTAGCC
TTATTTAAACTTGCTATGCTGTTTCCAGCATAGCTCTTAAACCCGTCTTCCCGAAGACCCTGCTCGATGCAGGGATCGAACCTGCGACCGTGGCGTTATTAGCACCACGCTCTGCCGACTGAGCTAA
TCGAGCCTCGAGTTTTTTAAATAAAAAATGGGTTTTTTTTAAGTAAAGTTATTTGAAATTGATTGAAATTTTCAAACCATGGGTGGTTTTTCGCTTTAAAATTGGGATTTTATTTTTATTTTTTTAT
ATTTTTTATTTTTTATTTTTTTTTTTTTGAGGTTTCTGAGATTATAAAATGAAATTTTTTTTTTTTTTTTAATTAATTCAAAAAATAATCAAATAAATAAATATAATATAAAAGATCCAAAATGGATG
GTGAAGATGTTCAAGCTGGATCGTTTCGCATGATTGAACAAGATGGATTGCACGCAGGTTCTCCGGCCGCTTGGGTGGAGAGGCTATTCGGCTATGACTGGGCACAACAGACAATCGGCTGCTCTGA
TGCCGCCGTGTTCCGGCTGTCAGCGCAGGGGCGCCCGGTTCTTTTTGTCAAGACCGACCTGTCCGGTGCCCTGAATGAACTGCAGGACGAGGCAGCGCGGCTATCGTGGCTGGCCACGACGGGCGTT
CCTTGCGCAGCTGTGCTCGACGTTGTCACTGAAGCGGGAAGGGACTGGCTGCTATTGGGCGAAGTGCCGGGGCAGGATCTCCTGTCATCTCACCTTGCTCCTGCCGAGAAAGTATCCATCATGGCTG
ATGCAATGCGGCGGCTGCATACGCTTGATCCGGCTACCTGCCCATTCGACCACCAAGCGAAACATCGCATCGAGCGAGCACGTACTCGGATGGAAGCCGGTCTTGTCGATCAGGATGATCTGGACGA
AGAGCATCAGGGGCTCGCGCCAGCCGAACTGTTCGCCAGGCTCAAGGCGCGCATGCCCGACGGCGAGGATCTCGTCGTGACCCATGGCGATGCCTGCTTGCCGAATATCATGGTGGAAAATGGCCGC
TTTTCTGGATTCATCGACTGTGGACGGCTGGGTGTGGCGGACCGCTATCAGGACATAGCGTTGGCTACCCGTGATATTGCTGAAGAGCTTGGCGGCGAATGGGCTGACCGCTTCCTCGTGCTTTACG
GTATCGCCGCTCCCGATTCGCAGCGCATCGCCTTCTATCGCCTTCTTGACGAGTTCTTCTGAACTAGAATCAATTTGATTTCTTCTTAATTCAATGCTAAAAAAAAAAATATTATAAAAAAAAAAAC
ACACACAACAAATAAATTAATAAATACCATTTATTAAAAAAAAAAAAAAAAAAAAAAAACCTATTTTAACAATTATTTTATTATTTTTATTTTTAGTTTGTTAAAAAAAAAATTATTATCTTTATT
TATAATTATAAAAAATTTATCCAATGATCATTTTTTAACTTTTTTATTATTTAGTATATTAAATAAAATGGATCCACTAGTTCTAGAGCGGCCGCCACCGCGGTGGAGCTCCAGCTTTTGTTCCCTT
TAGTGAGGGTTAATTGCGCGCTTGGCGTAATCATGGTCATAGCTGTTTCCTGTGTGAAATTGTTATCCGCTCACAATTCCACACAACATACGAGCCGGAAGCATAAAGTGTAAAGCCTGGGGTGCCT
AATGAGTGAGCTAACTCACATTAATTGCGTTGCGCTCACTGCCCGCTTTCCAGTCGGGAAACCTGTCGTGCCAGCTGCATTAATGAATCGGCCAACGCGCGGGGAGAGGCGGTTTGCGTATTGGGCG
CTCTTCCGCTTCCTCGCTCACTGACTCGCTGCGCTCGGTCGTTCGGCTGCGGCGAGCGGTATCAGCTCACTCAAAGGCGGTAATACGGTTATCCACAGAATCAGGGGATAACGCAGGAAAGAACATG
TGAGCAAAAGGCCAGCAAAAGGCCAGGAACCGTAAAAAGGCCGCGTTGCTGGCGTTTTTCCATAGGCTCCGCCCCCCTGACGAGCATCACAAAAATCGACGCTCAAGTCAGAGGTGGCGAAACCCGA
CAGGACTATAAAGATACCAGGCGTTTCCCCCTGGAAGCTCCCTCGTGCGCTCTCCTGTTCCGACCCTGCCGCTTACCGGATACCTGTCCGCCTTTCTCCCTTCGGGAAGCGTGGCGCTTTCTCATAG
CTCACGCTGTAGGTATCTCAGTTCGGTGTAGGTCGTTCGCTCCAAGCTGGGCTGTGTGCACGAACCCCCCGTTCAGCCCGACCGCTGCGCCTTATCCGGTAACTATCGTCTTGAGTCCAACCCGGTA
AGACACGACTTATCGCCACTGGCAGCAGCCACTGGTAACAGGATTAGCAGAGCGAGGTATGTAGGCGGTGCTACAGAGTTCTTGAAGTGGTGGCCTAACTACGGCTACACTAGAAGGACAGTATTTG
GTATCTGCGCTCTGCTGAAGCCAGTTACCTTCGGAAAAAGAGTTGGTAGCTCTTGATCCGGCAAACAAACCACCGCTGGTAGCGGTGGTTTTTTTGTTTGCAAGCAGCAGATTACGCGCAGAAAAAA
AGGATCTCAAGAAGATCCTTTGATCTTTTCTACGGGGTCTGACGCTCAGTGGAACGAAAACTCACGTTAAGGGATTTTGGTCATGAGATTATCAAAAAGGATCTTCACCTAGATCCTTTTAAATTAA
AAATGAAGTTTTAAATCAATCTAAAGTATATATGAGTAAACTTGGTCTGACAGTTACCAATGCTTAATCAGTGAGGCACCTATCTCAGCGATCTGTCTATTTCGTTCATCCATAGTTGCCTGACTCC
CCGTCGTGTAGATAACTACGATACGGGAGGGCTTACCATCTGGCCCCAGTGCTGCAATGATACCGCGAGACCCACGCTCACCGGCTCCAGATTTATCAGCAATAAACCAGCCAGCCGGAAGGGCCGA
GCGCAGAAGTGGTCCTGCAACTTTATCCGCCTCCATCCAGTCTATTAATTGTTGCCGGGAAGCTAGAGTAAGTAGTTCGCCAGTTAATAGTTTGCGCAACGTTGTTGCCATTGCTACAGGCATCGTG
GTGTCACGCTCGTCGTTTGGTATGGCTTCATTCAGCTCCGGTTCCCAACGATCAAGGCGAGTTACATGATCCCCCATGTTGTGCAAAAAAGCGGTTAGCTCCTTCGGTCCTCCGATCGTTGTCAGAA
GTAAGTTGGCCGCAGTGTTATCACTCATGGTTATGGCAGCACTGCATAATTCTCTTACTGTCATGCCATCCGTAAGATGCTTTTCTGTGACTGGTGAGTACTCAACCAAGTCATTCTGAGAATAGTG
TATGCGGCGACCGAGTTGCTCTTGCCCGGCGTCAATACGGGATAATACCGCGCCACATAGCAGAACTTTAAAAGTGCTCATCATTGGAAAACGTTCTTCGGGGCGAAAACTCTCAAGGATCTTACCG
CTGTTGAGATCCAGTTCGATGTAACCCACTCGTGCACCCAACTGATCTTCAGCATCTTTTACTTTCACCAGCGTTTCTGGGTGAGCAAAAACAGGAAGGCAAAATGCCGCAAAAAAGGGAATAAGGG
CGACACGGAAATGTTGAATACTCATACTCTTCCTTTTTCAATATTATTGAAGCATTTATCAGGGTTATTGTCTCATGAGCGGATACATATTTGAATGTATTTAGAAAAATAAACAAATAGGGGTTCC
GCGCACATTTCCCCGAAAAGTGCCAC
```

Supplementary Figure S6. (*continued*)

```
>pTM1599(SpCas9-NLS)
CTAAATTGTAAGCGTTAATATTTTGTTAAAATTCGCGTTAAATTTTTGTTAAATCAGCTCATTTTTTAACCAATAGGCCGAAATCGGCAAAATCCCTTATAAATCAAAAGAATAGACCGAGATAGGG
TTGAGTGTTGTTCCAGTTTGGAACAAGAGTCCACTATTAAAGAACGTGGACTCCAACGTCAAAGGGCGAAAAACCGTCTATCAGGGCGATGGCCCACTACGTGAACCATCACCCTAATCAAGTTTTT
TGGGGTCGAGGTGCCGTAAAGCACTAAATCGGAACCCTAAAGGGAGCCCCCGATTTAGAGCTTGACGGGGAAAGCCGGCGAACGTGGCGAGAAAGGAAGGGAAGAAAGCGAAAGGAGCGGGCGCTAG
GGCGCTGGCAAGTGTAGCGGTCACGCTGCGCGTAACCACCACACCCGCCGCGCTTAATGCGCCGCTACAGGGCGCGTCCCATTCGCCATTCAGGCTGCGCAACTGTTGGGAAGGGCGATCGGTGCGG
GCCTCTTCGCTATTACGCCAGCTGGCGAAAGGGGGATGTGCTGCAAGGCGATTAAGTTGGGTAACGCCAGGGTTTTCCCAGTCACGACGTTGTAAAACGACGGCCAGTGAGCGCGCGTAATACGACT
CACTATAGGGCGAATTGGGTACCGGGCCCCCCCTCGAGACTAGATAAAAAAAATTTTTATTTATTTTTATTTATTTTGAATTAAATAGATTACAAATTAATTAATCCCATCAAATCTTTAAAAAAAA
AAATGGTTTAAAAAACTTGGGTTGGTTAATTATTATTTGAAAATTTTAAAACCCAAATTAAAAAAAAAAAAATGGGATTCAAAAATTTTTTTTTTTTTTTTTTTTTTTTTTTTTTTTTTCAGATTGCA
TAAAAAGATTTTTTTTTTTTTTTTTTTCTTATTTCTTAAAACAAATAAATTAAATTAAATAAAAAATAAAAATCAGATCTATACAACAATAAAATGGATAAAAAATATTCAATTGGTTTAGATATTG
GTACAAATAGTGTTGGTTGGGCAGTTATTACAGATGAATATAAAGTTCCATCAAAAAAATTTAAAGTTTTAGGTAATACAGACCGGCATTCCATCAAGAAAAATCTGATCGGAGCTCTCCTCTTTGA
TTCAGGGGAGACCGCTGAAGCAACCCGCCTCAAGCGGACTGCTAGACGGCGGTACACCAGGAGGAAGAACCGGATTTGTTACCTTCAAGAGATATTCTCCAACGAAATGGCAAAGGTCGACGACAGC
TTCTTCCATAGGCTGGAAGAATCATTCCTCGTGGAAGAGGATAAGAAGCATGAACGGCATCCCATCTTCGGTAATATCGTCGACGAGGTGGCCTATCACGAGAAATACCCAACCATCTACCATCTTC
GCAAAAAGCTGGTGGACTCAACCGACAAGGCAGACCTCCGGCTTATCTACCTGGCCCTGGCCCACATGATCAAGTTCAGAGGCCACTTCCTGATCGAGGGCGACCTCAATCCTGACAATAGCGATGT
GGATAAACTGTTCATCCAGCTGGTGCAGACTTACAACCAGCTCTTTGAAGAGAACCCCATCAATGCAAGCGGAGTCGATGCCAAGGCCATTCTGTCAGCCCGGCTGTCAAAGAGCCGCAGACTTGAG
AATCTTATCGCTCAGCTGCCGGGTGAAAAGAAAAATGGACTGTTCGGGAACCTGATTGCTCTTTCACTTGGGCTGACTCCCAATTTCAAGTCTAATTTCGACCTGGCAGAGGATGCCAAGCTGCAAC
TGTCCAAGGACACCTATGATGACGATCTCGACAACCTCCTGGCCCAGATCGGTGACCAATACGCCGACCTTTTCCTTGCTGCTAAGAATCTTTCTGACGCCATCCTGCTGTCTGACATTCTCCGCGT
GAACACTGAAATCACCAAGGCCCCTCTTTCAGCTTCAATGATTAAGCGGTATGATGAGCACCACCAGGACCTGACCCTGCTTAAGGCACTCGTCCGGCAGCAGCTTCCGGAGAAGTACAAGGAAATC
TTCTTTGACCAGTCAAAGAATGGATACGCCGGCTACATCGACGGAGGTGCCTCCCAAGAGGAATTTTATAAGTTTATCAAACCTATCCTTGAGAAGATGGACGGCACCGAAGAGCTCCTCGTGAAAC
TGAATCGGGAGGATCTGCTGCGGAAGCAGCGCACTTTCGACAATGGGAGCATTCCCCACCAGATCCATCTTGGGGAGCTTCACGCCATCCTTCGGCGCCAAGAGGACTTCTACCCCTTTCTTAAGGA
CAACAGGGAGAAGATTGAGAAAATTCTCACTTTCCGCATCCCCTACTACGTGGGACCCCTCGCCAGAGGAAATAGCCGGTTTGCTTGGATGACCAGAAAGTCAGAAGAAACTATCACTCCCTGGAAC
TTCGAAGAGGTGGTGGACAAGGGAGCCAGCGCTCAGTCATTCATCGAACGGATGACTAACTTCGATAAGAACCTCCCCAATGAGAAGGTCCTGCCGAAACATTCCCTGCTCTACGAGTACTTTACCG
TGTACAACGAGCTGACCAAGGTGAAATATGTCACCGAAGGGATGAGGAAGCCCGCATTCCTGTCAGGCGAACAAAAGAAGGCAATTGTGGACCTTCTGTTCAAGACCAATAGAAAGGTGACCGTGAA
GCAGCTGAAGGAGGACTATTTCAAGAAAATTGAATGCTTCGACTCTGTGGAGATTAGCGGGGTCGAAGATCGGTTCAACGCAAGCCTGGGTACCTACCATGATCTGCTTAAGATCATCAAGGACAAG
GATTTTCTGGACAATGAGGAGAACGAGGACATCCTTGAGGACATTGTCCTGACTCTCACTCTGTTCGAGGACCGGGAAATGATCGAGGAGAGGCTTAAGACCTACGCCCATCTGTTCGACGATAAAG
TGATGAAGCAACTTAAACGGAGAAGATATACCGGATGGGGACGCCTTAGCCGCAAACTCATCAACGGAATCCGGGACAAACAGAGCGGAAAGACCATTCTTGATTTCCTTAAGAGCGACGGATTCGC
TAATCGCAACTTCATGCAACTTATCCATGATGATTCCCTGACCTTTAAGGAGGACATCCAGAAGGCCCAAGTGTCTGGACAAGGTGACTCACTGCACGAGCATATCGCAAATCTGGCTGGTTCACCC
GCTATTAAGAAGGGTATTCTCCAGACCGTGAAAGTCGTGGACGAGCTGGTCAAGGTGATGGGTCGCCATAAACCAGAGAACATTGTCATCGAGATGGCCAGGGAAAACCAGACTACCCAGAAGGGAC
AGAAGAACAGCAGGGAGCGGATGAAAAGAATTGAGGAAGGGATTAAGGAGCTCGGGTCACAGATCCTTAAAGAGCACCCGGTGGAAAACACCCAGCTTCAGAATGAGAAGCTCTATCTGTACTACCT
TCAAAATGGACGCGATATGTATGTGGACCAAGAGCTTGATATCAACAGGCTCTCAGACTACGACGTGGACCATATCGTCCCTCAGAGCTTCCTCAAAGACGACTCAATTGACAATAAGGTGCTGACT
CGCTCAGACAAGAACCGGGGAAAGTCAGATAACGTGCCCTCAGAGGAAGTCGTGAAAAAGATGAAGAACTATTGGCGCCAGCTTCTGAACGCAAAGCTGATCACTCAGCGGAAGTTCGACAATCTCA
CTAAGGCTGAGAGGGGCGGACTGAGCGAACTGGACAAAGCAGGATTCATTAAACGGCAACTTGTGGAGACTCGGCAGATTACTAAACATGTCGCCCAAATCCTTGACTCACGCATGAATACCAAGTA
CGACGAAAACGACAAACTTATCCGCGAGGTGAAGGTGATTACCCTGAAGTCCAAGCTGGTCAGCGATTTCAGAAAGGACTTTCAATTCTACAAAGTGCGGGAGATCAATAACTATCATCATGCTCAT
GACGCATATCTGAATGCCGTGGTGGGAACCGCCCTGATCAAGAAGTACCCAAAGCTGGAAAGCGAGTTCGTGTACGGAGACTACAAGGTCTACGACGTGCGCAAGATGATTGCCAAATCTGAGCAGG
AGATCGGAAAGGCCACCGCAAAGTACTTCTTCTACAGCAACATCATGAATTTCTTCAAGACCGAAATCACCCTTGCAAACGGTGAGATCCGGAAGAGGCCGCTCATCGAGACTAATGGGGAGACTGG
CGAAATCGTGTGGGACAAGGGCAGAGATTTCGCTACCGTGCGCAAAGTGCTTTCTATGCCTCAAGTGAACATCGTGAAGAAAACCGAGGTGCAAACCGGAGGCTTTTCTAAGGAATCAATCCTCCCC
AAGCGCAACTCCGACAAGCTCATTGCAAGGAAGAAGGATTGGGACCCTAAGAAGTACGGCGGATTCGATTCACCAACTGTGGCTTATTCTGTCCTGGTCGTGGCTAAGGTGGAAAAAGGAAAGTCTA
AGAAGCTCAAGAGCGTGAAGGAACTGCTGGGTATCACCATTATGGAGCGCAGCTCCTTCGAGAAGAACCCAATTGACTTTCTCGAAGCCAAAGGTTACAAGGAAGTCAAGAAGGACCTTATCATCAA
GCTCCCAAAGTATAGCCTGTTCGAACTGGAGAATGGGCGGAAGCGGATGCTCGCCTCCGCTGGCGAACTTCAGAAGGGTAATGAGCTGGCTCTCCCCTCCAAGTACGTGAATTTCCTCTACCTTGCA
AGCCATTACGAGAAGCTGAAGGGGAGCCCCGAGGACAACGAGCAAAAGCAACTGTTTGTGGAGCAGCATAAGCATTATCTGGACGAGATCATTGAGCAGATTTCCGAGTTTTCTAAACGCGTCATTC
TCGCTGATGCCAACCTCGATAAAGTCCTTAGCGCATACAATAAGCACAGAGACAAACCAATTCGGGAGCAGGCTGAGAATATCATCCACCTGTTCACCCTCACCAATCTTGGTGCCCCTGCCGCATT
CAAGTACTTCGACACCACCATCGACCGGAAACGCTATACCTCCACCAAAGAAGTGCTGGACGCCACCCTCATCCACCAGAGCATCACCGGACTTTACGAAACTCGGATTGACCTCTCACAGCTCGGA
GGGGATGAGGGAGCTCCCAAGAAAAAGCGCAAGGTAGGTAGTTCCATCGCTAGTTAAATAAATAAATTATTTAATAAATAATAAAAAAACAAATTGTTGTAATAATCTAATATTTTCTTTTTTTTTT
AATTTTTTTTTTTTAAATCTTAATAATTATTAAGTTATTTTAATTTTTTTTTTTTTTTTTTTTTTTTTTTTTTTTTTTTTTCTATCAAAAAAATCAAATATATTTAAAAAATTTATTATTTACAGATACA
TTTTGAATGGTGAAGATAAATATATGCATTAGATGTAAAACAGCCAAAGAGTATGAAAATCAAAAAGATAAAGCTTAAAAAAAGCACCGACTCGGTGCCACTTTTTCAAGTTGATAACGGACTAGCC
TTATTTAAACTTGCTATGCTGTTTCCAGCATAGCTCTTAAACCCGTCTTCCCGAAGACCCTGCTCGATGCAGGGATCGAACCTGCGACCGTGGCGTTATTAGCACCACGCTCTGCCGACTGAGCTAA
TCGAGCCTCGAGTTTTTTAAATAAAAAAATGGGTTTTTTTTAAGTAAAGTTATTTGAAATTGATTGAAATTTTCAAACCATGGGTGGTTTTTCGCTTTAAAATTGGGATTTTATTTTTATTTTTTTAT
ATTTTTTATTTTTTATTTTTTTTTTTTTTGAGGTTTCTGAGATTATAAAATGAAATTTTTTTTTTTTTTTAATTAATTCAAAAAATAATCAAATAAATAAATATAATATAAAAGATCCAAAATGGATG
GTGAAGATGTTCAAGCTGGATCGTTTCGCATGATTGAACAAGATGGATTGCACGCAGGTTCTCCGGCCGCTTGGGTGGAGAGGCTATTCGGCTATGACTGGGCACAACAGACAATCGGCTGCTCTGA
TGCCGCCGTGTTCCGGCTGTCAGCGCAGGGGCGCCCGGTTCTTTTTGTCAAGACCGACCTGTCCGGTGCCCTGAATGAACTGCAGGACGAGGCAGCGCGGCTATCGTGGCTGGCCACGACGGGCGTT
CCTTGCGCAGCTGTGCTCGACGTTGTCACTGAAGCGGGAAGGGACTGGCTGCTATTGGGCGAAGTGCCGGGGCAGGATCTCCTGTCATCTCACCTTGCTCCTGCCGAGAAAGTATCCATCATGGCTG
ATGCAATGCGGCGGCTGCATACGCTTGATCCGGCTACCTGCCCATTCGACCACCAAGCGAAACATCGCATCGAGCGAGCACGTACTCGGATGGAAGCCGGTCTTGTCGATCAGGATGATCTGGACGA
AGAGCATCAGGGGCTCGCGCCAGCCGAACTGTTCGCCAGGCTCAAGGCGCGCATGCCCGACGGCGAGGATCTCGTCGTGACCCATGGCGATGCCTGCTTGCCGAATATCATGGTGGAAAATGGCCGC
TTTTCTGGATTCATCGACTGTGGACGGCTGGGTGTGGCGGACCGCTATCAGGACATAGCGTTGGCTACCCGTGATATTGCTGAAGAGCTTGGCGGCGAATGGGCTGACCGCTTCCTCGTGCTTTACG
GTATCGCCGCTCCCGATTCGCAGCGCATCGCCTTCTATCGCCTTCTTGACGAGTTCTTCTGAACTAGAATCAATTTGATTTCTTCTTAATTCAATGCTAAAAAAAAAAATATTATAAAAAAAAAAAC
ACACACAACAAATAAATTAATAAATACCATTTATTAAAAAAAAAAAAAAAAAAAAAAAACCTATTTTAACAATTATTTTATTATTTTTATTTTTAGTTTTGTTAAAAAAAAAATTATTATCTTTATT
TATAATTATAAAAAATTTATCCAATGATCATTTTTTAACTTTTTTATTATTTAGTATATTAAATAAAATGGATCCACTAGTTCTAGAGCGGCCGCCACCGCGGTGGAGCTCCAGCTTTTGTTCCCTT
TAGTGAGGGTTAATTGCGCGCTTGGCGTAATCATGGTCATAGCTGTTTCCTGTGTGAAATTGTTATCCGCTCACAATTCCACACAACATACGAGCCGGAAGCATAAAGTGTAAAGCCTGGGGTGCCT
AATGAGTGAGCTAACTCACATTAATTGCGTTGCGCTCACTGCCCGCTTTCCAGTCGGGAAACCTGTCGTGCCAGCTGCATTAATGAATCGGCCAACGCGCGGGGAGAGGCGGTTTGCGTATTGGGCG
CTCTTCCGCTTCCTCGCTCACTGACTCGCTGCGCTCGGTCGTTCGGCTGCGGCGAGCGGTATCAGCTCACTCAAAGGCGGTAATACGGTTATCCACAGAATCAGGGGATAACGCAGGAAAGAACATG
TGAGCAAAAGGCCAGCAAAAGGCCAGGAACCGTAAAAAGGCCGCGTTGCTGGCGTTTTTCCATAGGCTCCGCCCCCCTGACGAGCATCACAAAAATCGACGCTCAAGTCAGAGGTGGCGAAACCCGA
CAGGACTATAAAGATACCAGGCGTTTCCCCCTGGAAGCTCCCTCGTGCGCTCTCCTGTTCCGACCCTGCCGCTTACCGGATACCTGTCCGCCTTTCTCCCTTCGGGAAGCGTGGCGCTTTCTCATAG
CTCACGCTGTAGGTATCTCAGTTCGGTGTAGGTCGTTCGCTCCAAGCTGGGCTGTGTGCACGAACCCCCCGTTCAGCCCGACCGCTGCGCCTTATCCGGTAACTATCGTCTTGAGTCCAACCCGGTA
AGACACGACTTATCGCCACTGGCAGCAGCCACTGGTAACAGGATTAGCAGAGCGAGGTATGTAGGCGGTGCTACAGAGTTCTTGAAGTGGTGGCCTAACTACGGCTACACTAGAAGGACAGTATTTG
GTATCTGCGCTCTGCTGAAGCCAGTTACCTTCGGAAAAAGAGTTGGTAGCTCTTGATCCGGCAAACAAACCACCGCTGGTAGCGGTGGTTTTTTTGTTTGCAAGCAGCAGATTACGCGCAGAAAAAA
AGGATCTCAAGAAGATCCTTTGATCTTTTCTACGGGGTCTGACGCTCAGTGGAACGAAAACTCACGTTAAGGGATTTTGGTCATGAGATTATCAAAAAGGATCTTCACCTAGATCCTTTTAAATTAA
AAATGAAGTTTTAAATCAATCTAAAGTATATATGAGTAAACTTGGTCTGACAGTTACCAATGCTTAATCAGTGAGGCACCTATCTCAGCGATCTGTCTATTTCGTTCATCCATAGTTGCCTGACTCC
CCGTCGTGTAGATAACTACGATACGGGAGGGCTTACCATCTGGCCCCAGTGCTGCAATGATACCGCGAGACCCACGCTCACCGGCTCCAGATTTATCAGCAATAAACCAGCCAGCCGGAAGGGCCGA
GCGCAGAAGTGGTCCTGCAACTTTATCCGCCTCCATCCAGTCTATTAATTGTTGCCGGGAAGCTAGAGTAAGTAGTTCGCCAGTTAATAGTTTGCGCAACGTTGTTGCCATTGCTACAGGCATCGTG
GTGTCACGCTCGTCGTTTGGTATGGCTTCATTCAGCTCCGGTTCCCAACGATCAAGGCGAGTTACATGATCCCCCATGTTGTGCAAAAAAGCGGTTAGCTCCTTCGGTCCTCCGATCGTTGTCAGAA
GTAAGTTGGCCGCAGTGTTATCACTCATGGTTATGGCAGCACTGCATAATTCTCTTACTGTCATGCCATCCGTAAGATGCTTTTCTGTGACTGGTGAGTACTCAACCAAGTCATTCTGAGAATAGTG
TATGCGGCGACCGAGTTGCTCTTGCCCGGCGTCAATACGGGATAATACCGCGCCACATAGCAGAACTTTAAAAGTGCTCATCATTGGAAAACGTTCTTCGGGGCGAAAACTCTCAAGGATCTTACCG
CTGTTGAGATCCAGTTCGATGTAACCCACTCGTGCACCCAACTGATCTTCAGCATCTTTTACTTTCACCAGCGTTTCTGGGTGAGCAAAAACAGGAAGGCAAAATGCCGCAAAAAAGGGAATAAGGG
CGACACGGAAATGTTGAATACTCATACTCTTCCTTTTTCAATATTATTGAAGCATTTATCAGGGTTATTGTCTCATGAGCGGATACATATTTGAATGTATTTAGAAAAATAAACAAATAGGGGTTCC
GCGCACATTTCCCCGAAAAGTGCCAC
```

Supplementary Figure S6. (*continued*)

```
>pTM1644(SpCas9-NLS Esp3I)
CTAAATTGTAAGCGTTAATATTTTGTTAAAATTCGCGTTAAATTTTTGTTAAATCAGCTCATTTTTTAACCAATAGGCCGAAATCGGCAAAATCCCTTATAAATCAAAAGAATAGACCGAGATAGGG
TTGAGTGTTGTTCCAGTTTGGAACAAGAGTCCACTATTAAAGAACGTGGACTCCAACGTCAAAGGGCGAAAAACCGTCTATCAGGGCGATGGCCCACTACGTGAACCATCACCCTAATCAAGTTTTT
TGGGGTCGAGGTGCCGTAAAGCACTAAATCGGAACCCTAAAGGGAGCCCCCGATTTAGAGCTTGACGGGGAAAGCCGGCGAACGTGGCGAGAAAGGAAGGGAAGAAAGCGAAAGGAGCGGGCGCTAG
GGCGCTGGCAAGTGTAGCGGTCACGCTGCGCGTAACCACCACACCCGCCGCGCTTAATGCGCCGCTACAGGGCGCGTCCCATTCGCCATTCAGGCTGCGCAACTGTTGGGAAGGGCGATCGGTGCGG
GCCTCTTCGCTATTACGCCAGCTGGCGAAAGGGGGATGTGCTGCAAGGCGATTAAGTTGGGTAACGCCAGGGTTTTCCCAGTCACGACGTTGTAAAACGACGGCCAGTGAGCGCGCGTAATACGACT
CACTATAGGGCGAATTGGGTACCGGGCCCCCCCTCGAGACTAGATAAAAAAAATTTTTATTTATTTTTATTTATTTTGAATTAAATAGATTACAAATTAATTAATCCCATCAAATCTTTAAAAAAAA
AAATGGTTTAAAAAACTTGGGTTGGTTAATTATTATTTGAAAATTTTAAAACCCAAATTAAAAAAAAAAAAATGGGATTCAAAAATTTTTTTTTTTTTTTTTTTTTTTTTTTTTTTTTTTCAGATTGCA
TAAAAAGATTTTTTTTTTTTTTTTTTTCTTATTTCTTAAAACAAATAAATTAAATTAAATAAAAAATAAAAATCAGATCTATACAACAATAAAATGGATAAAAAATATTCAATTGGTTTAGATATTG
GTACAAATAGTGTTGGTTGGGCAGTTATTACAGATGAATATAAAGTTCCATCAAAAAAATTTAAAGTTTTAGGTAATACAGACCGGCATTCCATCAAGAAAAATCTGATCGGAGCTCTCCTCTTTGA
TTCAGGGGAGACCGCTGAAGCAACCCGCCTCAAGCGGACTGCTAGACGGCGGTACACCAGGAGGAAGAACCGGATTTGTTACCTTCAAGAGATATTCTCCAACGAAATGGCAAAGGTCGACGACAGC
TTCTTCCATAGGCTGGAAGAATCATTCCTCGTGGAAGAGGATAAGAAGCATGAACGGCATCCCATCTTCGGTAATATCGTCGACGAGGTGGCCTATCACGAGAAATACCCAACCATCTACCATCTTC
GCAAAAAGCTGGTGGACTCAACCGACAAGGCAGACCTCCGGCTTATCTACCTGGCCCTGGCCCACATGATCAAGTTCAGAGGCCACTTCCTGATCGAGGGCGACCTCAATCCTGACAATAGCGATGT
GGATAAACTGTTCATCCAGCTGGTGCAGACTTACAACCAGCTCTTTGAAGAGAACCCCATCAATGCAAGCGGAGTCGATGCCAAGGCCATTCTGTCAGCCCGGCTGTCAAAGAGCCGCAGACTTGAG
AATCTTATCGCTCAGCTGCCGGGTGAAAAGAAAAATGGACTGTTCGGGAACCTGATTGCTCTTTCACTTGGGCTGACTCCCAATTTCAAGTCTAATTTCGACCTGGCAGAGGATGCCAAGCTGCAAC
TGTCCAAGGACACCTATGATGACGATCTCGACAACCTCCTGGCCCAGATCGGTGACCAATACGCCGACCTTTTCCTTGCTGCTAAGAATCTTTCTGACGCCATCCTGCTGTCTGACATTCTCCGCGT
GAACACTGAAATCACCAAGGCCCCTCTTTCAGCTTCAATGATTAAGCGGTATGATGAGCACCACCAGGACCTGACCCTGCTTAAGGCACTCGTCCGGCAGCAGCTTCCGGAGAAGTACAAGGAAATC
TTCTTTGACCAGTCAAAGAATGGATACGCCGGCTACATCGACGGAGGTGCCTCCCAAGAGGAATTTTATAAGTTTATCAAACCTATCCTTGAGAAGATGGACGGCACCGAAGAGCTCCTCGTGAAAC
TGAATCGGGAGGATCTGCTGCGGAAGCAGCGCACTTTCGACAATGGGAGCATTCCCCACCAGATCCATCTTGGGGAGCTTCACGCCATCCTTCGGCGCCAAGAGGACTTCTACCCCTTTCTTAAGGA
CAACAGGGAGAAGATTGAGAAAATTCTCACTTTCCGCATCCCCTACTACGTGGGACCCCTCGCCAGAGGAAATAGCCGGTTTGCTTGGATGACCAGAAAGTCAGAAGAAACTATCACTCCCTGGAAC
TTCGAAGAGGTGGTGGACAAGGGAGCCAGCGCTCAGTCATTCATCGAACGGATGACTAACTTCGATAAGAACCTCCCCAATGAGAAGGTCCTGCCGAAACATTCCCTGCTCTACGAGTACTTTACCG
TGTACAACGAGCTGACCAAGGTGAAATATGTCACCGAAGGGATGAGGAAGCCCGCATTCCTGTCAGGCGAACAAAAGAAGGCAATTGTGGACCTTCTGTTCAAGACCAATAGAAAGGTGACCGTGAA
GCAGCTGAAGGAGGACTATTTCAAGAAAATTGAATGCTTCGACTCTGTGGAGATTAGCGGGGTCGAAGATCGGTTCAACGCAAGCCTGGGTACCTACCATGATCTGCTTAAGATCATCAAGGACAAG
GATTTTCTGGACAATGAGGAGAACGAGGACATCCTTGAGGACATTGTCCTGACTCTCACTCTGTTCGAGGACCGGGAAATGATCGAGGAGAGGCTTAAGACCTACGCCCATCTGTTCGACGATAAAG
TGATGAAGCAACTTAAACGGAGAAGATATACCGGATGGGGACGCCTTAGCCGCAAACTCATCAACGGAATCCGGGACAAACAGAGCGGAAAGACCATTCTTGATTTCCTTAAGAGCGACGGATTCGC
TAATCGCAACTTCATGCAACTTATCCATGATGATTCCCTGACCTTTAAGGAGGACATCCAGAAGGCCCAAGTGTCTGGACAAGGTGACTCACTGCACGAGCATATCGCAAATCTGGCTGGTTCACCC
GCTATTAAGAAGGGTATTCTCCAGACCGTGAAAGTCGTGGACGAGCTGGTCAAGGTGATGGGTCGCCATAAACCAGAGAACATTGTCATCGAGATGGCCAGGGAAAACCAGACTACCCAGAAGGGAC
AGAAGAACAGCAGGGAGCGGATGAAAAGAATTGAGGAAGGGATTAAGGAGCTCGGGTCACAGATCCTTAAAGAGCACCCGGTGGAAAACACCCAGCTTCAGAATGAGAAGCTCTATCTGTACTACCT
TCAAAATGGACGCGATATGTATGTGGACCAAGAGCTTGATATCAACAGGCTCTCAGACTACGACGTGGACCATATCGTCCCTCAGAGCTTCCTCAAAGACGACTCAATTGACAATAAGGTGCTGACT
CGCTCAGACAAGAACCGGGGAAAGTCAGATAACGTGCCCTCAGAGGAAGTCGTGAAAAAGATGAAGAACTATTGGCGCCAGCTTCTGAACGCAAAGCTGATCACTCAGCGGAAGTTCGACAATCTCA
CTAAGGCTGAGAGGGGCGGACTGAGCGAACTGGACAAAGCAGGATTCATTAAACGGCAACTTGTGGAGACTCGGCAGATTACTAAACATGTCGCCCAAATCCTTGACTCACGCATGAATACCAAGTA
CGACGAAAACGACAAACTTATCCGCGAGGTGAAGGTGATTACCCTGAAGTCCAAGCTGGTCAGCGATTTCAGAAAGGACTTTCAATTCTACAAAGTGCGGGAGATCAATAACTATCATCATGCTCAT
GACGCATATCTGAATGCCGTGGTGGGAACCGCCCTGATCAAGAAGTACCCAAAGCTGGAAAGCGAGTTCGTGTACGGAGACTACAAGGTCTACGACGTGCGCAAGATGATTGCCAAATCTGAGCAGG
AGATCGGAAAGGCCACCGCAAAGTACTTCTTCTACAGCAACATCATGAATTTCTTCAAGACCGAAATCACCCTTGCAAACGGTGAGATCCGGAAGAGGCCGCTCATCGAGACTAATGGGGAGACTGG
CGAAATCGTGTGGGACAAGGGCAGAGATTTCGCTACCGTGCGCAAAGTGCTTTCTATGCCTCAAGTGAACATCGTGAAGAAAACCGAGGTGCAAACCGGAGGCTTTTCTAAGGAATCAATCCTCCCC
AAGCGCAACTCCGACAAGCTCATTGCAAGGAAGAAGGATTGGGACCCTAAGAAGTACGGCGGATTCGATTCACCAACTGTGGCTTATTCTGTCCTGGTCGTGGCTAAGGTGGAAAAAGGAAAGTCTA
AGAAGCTCAAGAGCGTGAAGGAACTGCTGGGTATCACCATTATGGAGCGCAGCTCCTTCGAGAAGAACCCAATTGACTTTCTCGAAGCCAAAGGTTACAAGGAAGTCAAGAAGGACCTTATCATCAA
GCTCCCAAAGTATAGCCTGTTCGAACTGGAGAATGGGCGGAAGCGGATGCTCGCCTCCGCTGGCGAACTTCAGAAGGGTAATGAGCTGGCTCTCCCCTCCAAGTACGTGAATTTCCTCTACCTTGCA
AGCCATTACGAGAAGCTGAAGGGGAGCCCCGAGGACAACGAGCAAAAGCAACTGTTTGTGGAGCAGCATAAGCATTATCTGGACGAGATCATTGAGCAGATTTCCGAGTTTTCTAAACGCGTCATTC
TCGCTGATGCCAACCTCGATAAAGTCCTTAGCGCATACAATAAGCACAGAGACAAACCAATTCGGGAGCAGGCTGAGAATATCATCCACCTGTTCACCCTCACCAATCTTGGTGCCCCTGCCGCATT
CAAGTACTTCGACACCACCATCGACCGGAAACGCTATACCTCCACCAAAGAAGTGCTGGACGCCACCCTCATCCACCAGAGCATCACCGGACTTTACGAAACTCGGATTGACCTCTCACAGCTCGGA
GGGGATGAGGGAGCTCCCAAGAAAAAGCGCAAGGTAGGTAGTTCCATCGCTAGTTAAATAAATAAATTATTTAATAAATAATAAAAAAACAAATTGTTGTAATAATCTAATATTTTCTTTTTTTTTT
AATTTTTTTTTTTTAAATCTTAATAATTATTAAGTTATTTTAATTTTTTTTTTTTTTTTTTTTTTTTTTTTTTTTTTTTTTCTATCAAAAAAATCAAATATATTTAAAAAATTTATTATTTACAGATACA
TTTTGAATGGTGAAGATAAATATATGCATTAGATGTAAAACAGCCAAAGAGTATGAAAATCAAAAAGATAAAGCTTAAAAAAAGCACCGACTCGGTGCCACTTTTTCAAGTTGATAACGGACTAGCC
TTATTTAAACTTGCTATGCTGTTTCCAGCATAGCTCTTAAACCGAGACGCCCGTCTCCTGCTCGATGCAGGGATCGAACCTGCGACCGTGGCGTTATTAGCACCACGCTCTGCCGACTGAGCTAATC
GAGCCTCGAGTTTTTTAAATAAAAAATGGGTTTTTTTTAAGTAAAGTTATTTGAAATTGATTGAAATTTTCAAACCATGGGTGGTTTTTCGCTTTAAAATTGGGATTTTATTTTTATTTTTTTATAT
TTTTTATTTTTTATTTTTTTTTTTTTTGAGGTTTCTGAGATTATAAAATGAAATTTTTTTTTTTTTTTTAATTAATTCAAAAAATAATCAAATAAATAAATATAATATAAAAGATCCAAAATGGATGGT
GAAGATGTTCAAGCTGGATCGTTTCGCATGATTGAACAAGATGGATTGCACGCAGGTTCTCCGGCCGCTTGGGTGGAGAGGCTATTCGGCTATGACTGGGCACAACAGACAATCGGCTGCTCTGATG
CCGCCGTGTTCCGGCTGTCAGCGCAGGGGCGCCCGGTTCTTTTTGTCAAGACCGACCTGTCCGGTGCCCTGAATGAACTGCAGGACGAGGCAGCGCGGCTATCGTGGCTGGCCACGACGGGCGTTCC
TTGCGCAGCTGTGCTCGACGTTGTCACTGAAGCGGGAAGGGACTGGCTGCTATTGGGCGAAGTGCCGGGGCAGGATCTCCTGTCATCTCACCTTGCTCCTGCCGAGAAAGTATCCATCATGGCTGAT
GCAATGCGGCGGCTGCATACGCTTGATCCGGCTACCTGCCCATTCGACCACCAAGCGAAACATCGCATCGAGCGAGCACGTACTCGGATGGAAGCCGGTCTTGTCGATCAGGATGATCTGGACGAAG
AGCATCAGGGGCTCGCGCCAGCCGAACTGTTCGCCAGGCTCAAGGCGCGCATGCCCGACGGCGAGGATCTCGTCGTGACCCATGGCGATGCCTGCTTGCCGAATATCATGGTGGAAAATGGCCGCTT
TTCTGGATTCATCGACTGTGGACGGCTGGGTGTGGCGGACCGCTATCAGGACATAGCGTTGGCTACCCGTGATATTGCTGAAGAGCTTGGCGGCGAATGGGCTGACCGCTTCCTCGTGCTTTACGGT
ATCGCCGCTCCCGATTCGCAGCGCATCGCCTTCTATCGCCTTCTTGACGAGTTCTTCTGAACTAGAATCAATTTGATTTCTTCTTAATTCAATGCTAAAAAAAAAAATATTATAAAAAAAAAAACAC
ACACAACAAATAAATTAATAAATACCATTTATTAAAAAAAAAAAAAAAAAAAAAAAAACCTATTTAACAATTATTTTATTATTTTTATTTTTAGTTTTGTTAAAAAAAAAATTATTATCTTTATTTA
TAATTATAAAAAATTTATCCAATGATCATTTTTTAACTTTTTTATTATTTAGTATATTAAATAAAATGGATCCACTAGTTCTAGAGCGGCCGCCACCGCGGTGGAGCTCCAGCTTTTGTTCCCTTTA
GTGAGGGTTAATTGCGCGCTTGGCGTAATCATGGTCATAGCTGTTTCCTGTGTGAAATTGTTATCCGCTCACAATTCCACACAACATACGAGCCGGAAGCATAAAGTGTAAAGCCTGGGGTGCCTAA
TGAGTGAGCTAACTCACATTAATTGCGTTGCGCTCACTGCCCGCTTTCCAGTCGGGAAACCTGTCGTGCCAGCTGCATTAATGAATCGGCCAACGCGCGGGGAGAGGCGGTTTGCGTATTGGGCGCT
CTTCCGCTTCCTCGCTCACTGACTCGCTGCGCTCGGTCGTTCGGCTGCGGCGAGCGGTATCAGCTCACTCAAAGGCGGTAATACGGTTATCCACAGAATCAGGGGATAACGCAGGAAAGAACATGTG
AGCAAAAGGCCAGCAAAAGGCCAGGAACCGTAAAAAGGCCGCGTTGCTGGCGTTTTTCCATAGGCTCCGCCCCCCTGACGAGCATCACAAAAATCGACGCTCAAGTCAGAGGTGGCGAAACCCGACA
GGACTATAAAGATACCAGGCGTTTCCCCCTGGAAGCTCCCTCGTGCGCTCTCCTGTTCCGACCCTGCCGCTTACCGGATACCTGTCCGCCTTTCTCCCTTCGGGAAGCGTGGCGCTTTCTCATAGCT
CACGCTGTAGGTATCTCAGTTCGGTGTAGGTCGTTCGCTCCAAGCTGGGCTGTGTGCACGAACCCCCCGTTCAGCCCGACCGCTGCGCCTTATCCGGTAACTATCGTCTTGAGTCCAACCCGGTAAG
ACACGACTTATCGCCACTGGCAGCAGCCACTGGTAACAGGATTAGCAGAGCGAGGTATGTAGGCGGTGCTACAGAGTTCTTGAAGTGGTGGCCTAACTACGGCTACACTAGAAGGACAGTATTTGGT
ATCTGCGCTCTGCTGAAGCCAGTTACCTTCGGAAAAAGAGTTGGTAGCTCTTGATCCGGCAAACAAACCACCGCTGGTAGCGGTGGTTTTTTTGTTTGCAAGCAGCAGATTACGCGCAGAAAAAAAG
GATCTCAAGAAGATCCTTTGATCTTTTCTACGGGGTCTGACGCTCAGTGGAACGAAAACTCACGTTAAGGGATTTTGGTCATGAGATTATCAAAAAGGATCTTCACCTAGATCCTTTTAAATTAAAA
ATGAAGTTTTAAATCAATCTAAAGTATATATGAGTAAACTTGGTCTGACAGTTACCAATGCTTAATCAGTGAGGCACCTATCTCAGCGATCTGTCTATTTCGTTCATCCATAGTTGCCTGACTCCCC
GTCGTGTAGATAACTACGATACGGGAGGGCTTACCATCTGGCCCCAGTGCTGCAATGATACCGCGAGACCCACGCTCACCGGCTCCAGATTTATCAGCAATAAACCAGCCAGCCGGAAGGGCCGAGC
GCAGAAGTGGTCCTGCAACTTTATCCGCCTCCATCCAGTCTATTAATTGTTGCCGGGAAGCTAGAGTAAGTAGTTCGCCAGTTAATAGTTTGCGCAACGTTGTTGCCATTGCTACAGGCATCGTGGT
GTCACGCTCGTCGTTTGGTATGGCTTCATTCAGCTCCGGTTCCCAACGATCAAGGCGAGTTACATGATCCCCCATGTTGTGCAAAAAAGCGGTTAGCTCCTTCGGTCCTCCGATCGTTGTCAGAAGT
AAGTTGGCCGCAGTGTTATCACTCATGGTTATGGCAGCACTGCATAATTCTCTTACTGTCATGCCATCCGTAAGATGCTTTTCTGTGACTGGTGAGTACTCAACCAAGTCATTCTGAGAATAGTGTA
TGCGGCGACCGAGTTGCTCTTGCCCGGCGTCAATACGGGATAATACCGCGCCACATAGCAGAACTTTAAAAGTGCTCATCATTGGAAAACGTTCTTCGGGGCGAAAACTCTCAAGGATCTTACCGCT
GTTGAGATCCAGTTCGATGTAACCCACTCGTGCACCCAACTGATCTTCAGCATCTTTTACTTTCACCAGCGTTTCTGGGTGAGCAAAAACAGGAAGGCAAAATGCCGCAAAAAAGGGAATAAGGGCG
ACACGGAAATGTTGAATACTCATACTCTTCCTTTTTCAATATTATTGAAGCATTTATCAGGGTTATTGTCTCATGAGCGGATACATATTTGAATGTATTTAGAAAAATAAACAAATAGGGGTTCCGC
GCACATTTCCCCGAAAAGTGCCAC
```

Supplementary Figure S6. (*continued*)

```
>pTM1668(SpRY-NLS)
CTAAATTGTAAGCGTTAATATTTTGTTAAAATTCGCGTTAAATTTTTGTTAAATCAGCTCATTTTTTAACCAATAGGCCGAAATCGGCAAAATCCCTTATAAATCAAAAGAATAGACCGAGATAGGG
TTGAGTGTTGTTCCAGTTTGGAACAAGAGTCCACTATTAAAGAACGTGGACTCCAACGTCAAAGGGCGAAAAACCGTCTATCAGGGCGATGGCCCACTACGTGAACCATCACCCTAATCAAGTTTTT
TGGGGTCGAGGTGCCGTAAAGCACTAAATCGGAACCCTAAAGGGAGCCCCCGATTTAGAGCTTGACGGGGAAAGCCGGCGAACGTGGCGAGAAAGGAAGGGAAGAAAGCGAAAGGAGCGGGCGCTAG
GGCGCTGGCAAGTGTAGCGGTCACGCTGCGCGTAACCACCACACCCGCCGCGCTTAATGCGCCGCTACAGGGCGCGTCCCATTCGCCATTCAGGCTGCGCAACTGTTGGGAAGGGCGATCGGTGCGG
GCCTCTTCGCTATTACGCCAGCTGGCGAAAGGGGGATGTGCTGCAAGGCGATTAAGTTGGGTAACGCCAGGGTTTTCCCAGTCACGACGTTGTAAAACGACGGCCAGTGAGCGCGCGTAATACGACT
CACTATAGGGCGAATTGGGTACCGGGCCCCCCCTCGAGACTAGATAAAAAAAATTTTATTTATTTTTATTTATTTTGAATTAAATAGATTACAAATTAATTAATCCCATCAAATCTTTAAAAAAAA
AAATGGTTTAAAAAACTTGGGTTGGTTAATTATTATTTGAAAATTTTAAAACCCAAATTAAAAAAAAAAAATGGGATTCAAAAATTTTTTTTTTTTTTTTTTTTTTTTTTTTTTTTTTTCAGATTGCA
TAAAAAGATTTTTTTTTTTTTTTTTTTCTTATTTCTTAAAACAAATAAATTAAATTAAATAAAAAATAAAAATCAGATCTATACAACAATAAAATGGATAAAAAATACTCTATAGGACTTGATATAG
GTACAAATAGTGTTGGATGGGCCGTAATTACAGATGAATACAAAGTACCTTCTAAAAAGTTTAAAGTTCTTGGAAATACTGATAGACACTCAATTAAGAAGAATTTGATAGGTGCCTTGTTATTCGA
TTCTGGAGAAACAGCTGAGAGAACACGTTTGAAGAGAACCGCTAGACGTCGTTACACTCGTCGTAAAAATCGTATTTGTTATTTACAAGAAATATTCAGTAATGAGATGGCAAAAGTAGATGATAGT
TTTTTCCATCGTTTAGAGGAATCATTCCTTGTAGAAGAGGATAAGAAGCATGAGCGTCATCCTATATTTGGAAATATAGTAGATGAGGTAGCCTACCACGAAAAGTATCCAACTATATATCACTTGA
GAAAGAAGTTAGTAGATTCAACTGATAAAGCCGATCTTCGTTTGATATATCTTGCCTTAGCTCATATGATTAAGTTCCGTGGACATTTCTTAATCGAAGGTGATTTGAATCCTGATAATTCAGATGT
AGATAAGTTGTTTATCCAACTTGTTCAAACATACAATCAATTATTTGAAGAGAATCCTATCAATGCCTCAGGAGTAGATGCAAAAGCTATCTTATCAGCACGTTTGAGTAAATCACGTCGTTTGGAA
AATCTTATTGCACAATTACCTGGTGAGAAGAAAAATGGTCTTTTCGGAAATCTTATCGCCTTATCTCTTGGACTTACACCTAATTTTAAGAGTAATTTTGATTTGGCTGAGGATGCCAAGTTGCAAT
TAAGTAAGGATACCTACGATGATGATCTTGATAATTTATTGGCCCAAATAGGAGATCAATATGCAGATTTGTTCCTTGCTGCAAAGAATTTATCTGATGCCATCCTTTTATCAGATATTTTGAGAGT
TAATACAGAGATTACTAAGGCCCCACTTTCTGCCAGTATGATTAAAAGATATGATGAGCATCACCAAGATTTAACTCTTTTGAAAGCTCTTGTTCGTCAACAATTACCAGAGAAGTACAAGGAGATA
TTCTTTGATCAAAGTAAGAATGGTTACGCTGGATACATTGATGGAGGTGCTTCTCAAGAAGAGTTCTACAAGTTTATTAAGCCTATACTTGAGAAAATGGATGGTACAGAGGAATTACTTGTTAAAT
TAAATAGAGAAGATTTACTTAGAAAGCAACGTACATTTGATAATGGATCTATTCCTCATCAAATTCACCTTGGAGAGTTACATGCCATATTGAGAAGACAAGAGGATTTCTATCCTTTCCTTAAAGA
TAATAGAGAGAAGATTGAGAAGATTTTAACCTTTCGTATTCCTTATTATGTAGGACCTTTGGCTCGTGGAAATAGTAGATTTGCATGGATGACAAGAAAATCAGAGGAAACTATCACCCCTTGGAAT
TTTGAAGAAGTTGTAGATAAAGGTGCATCTGCTCAAAGTTTTATCGAAAGAATGACTAATTTCGATAAGAATTTACCTAATGAGAAGGTACTTCCAAAACATTCATTATTATACGAGTATTTTACAG
TTTATAATGAGCTTACTAAGGTAAAGTATGTAACCGAAGGTATGAGAAAGCCTGCATTTCTTAGTGGAGAACAAAAAAAAGCTATTGTAGATTTGTTATTCAAGACCAATAGAAAAGTTACAGTAAA
GCAATTAAAGGAGGATTACTTCAAGAAGATTGAGTGTTTCGATAGTGTTGAGATTTCAGGAGTTGAGGATAGATTCAATGCTTCATTGGGTACCTACCACGATTTGTTGAAGATCATCAAGGATAAA
GATTTTCTTGATAATGAAGAAAATGAAGATATATTAGAAGATATAGTTTTAACTTTAACATTGTTCGAAGATCGTGAAATGATAGAGGAACGTTTGAAAACTTATGCCCATCTTTTCGATGATAAAG
TTATGAAACAATTGAAAAGAAGACGTTATACTGGTTGGGGACGTTTAAGTCGTAAGTTAATAAATGGTATTCGTGATAAGCAATCAGGTAAGACCATTTTAGATTTTTTGAAATCAGATGGATTTGC
TAATAGAAATTTCATGCAATTAATACACGATGATTCTTTAACTTTTAAGGAGGATATACAAAAAGCTCAAGTTTCTGGACAAGGAGATTCTTTACATGAACACATAGCAAATCTTGCTGGTTCACCT
GCAATAAAGAAAGGAATCTTGCAAACTGTTAAAGTTGTTGATGAATTGGTTAAGGTAATGGGTCGTCATAAACCTGAGAATATCGTTATCGAAATGGCCCGTGAAAATCAAACTACCCAAAAGGGAC
AAAAGAATAGTCGTGAACGTATGAAGCGTATCGAGGAAGGTATAAAGGAATTGGGATCTCAAATATTAAAGGAACATCCTGTAGAAAATACACAATTGCAAAATGAAAAGTTATATTTGTATTACTT
ACAAAATGGTCGTGATATGTATGTTGATCAAGAATTGGATATAAATAGATTATCTGATTATGATGTAGATCATATTGTTCCACAAAGTTTTTTAAAAGATGATTCTATTGATAATAAGGTTCTTACT
CGTTCTGATAAAAATCGTGGAAAGTCTGATAATGTTCCAAGTGAAGAAGTTGTAAAAAAGATGAAGAATTACTGGCGTCAATTATTAAATGCCAAATTGATAACACAACGTAAGTTTGATAATCTTA
CTAAAGCAGAGAGAGGTGGACTTTCAGAATTGGATAAGGCCGGATTTATAAAACGTCAATTAGTAGAGACCCGTCAAATTACTAAGCATGTAGCTCAAATACTTGATAGTAGAATGAATACCAAGTA
TGATGAAAATGATAAGTTAATTCGTGAAGTAAAGGTTATAACCCTTAAGAGTAAGTTAGTAAGTGATTTTCGTAAAGATTTCCAATTTTATAAAGTAAGAGAGATAAATAATTATCACCACGCCCAC
GATGCCTACTTAAATGCTGTAGTAGGTACTGCTCTTATAAAAAAATATCCTAAGTTGGAAAGTGAATTTGTATATGGAGATTATAAAGTATATGATGTTCGTAAAATGATCGCAAAGAGTGAGCAAG
AGATTGGTAAGGCTACAGCAAAGTACTTCTTCTATTCAAATATAATGAATTTCTTCAAGACAGAGATTACACTTGCTAATGGTGAAATACGTAAGCGTCCTTTAATTGAGACTAATGGTGAAACTGG
TGAAATAGTATGGGATAAGGGAAGAGATTTCGCTACTGTTCGTAAAGTACTTAGTATGCCACAAGTAAATATAGTTAAAAAAACTGAGGTACAAACTGGAGGTTTCTCTAAGGAGTCAATCCGTCCA
AAGCGTAATAGTGATAAATTGATAGCACGTAAAAAAGATTGGGACCCAAAGAAATATGGTGGTTTTTTATGGCCTACCGTTGCATATTCAGTTTTAGTAGTAGCAAAGGTTGAGAAAGGTAAATCAA
AAAAGTTGAAATCAGTTAAGGAGTTACTTGGAATTACAATAATGGAAAGATCATCTTTTGAGAAAAATCCTATTGATTTTTTGGAGGCAAAAGGATATAAAGAGGTTAAAAAGGATTTAATAATAAA
ATTGCCTAAGTACAGTCTTTTCGAATTAGAAAATGGTAGAAAACGTATGTTAGCCAGTGCTAAGCAATTGCAAAAGGGAAATGAACTTGCTCTTCCTTCAAAATACGTTAATTTTCTTTACCTTGCA
TCTCATTATGAGAAGTTAAAGGGTTCTCCAGAAGATAATGAGCAAAAACAATTGTTTGTAGAACAACACAAGCATTACTTGGATGAGATTATCGAACAAATATCAGAATTTTCAAAGAGAGTAATTT
TGGCAGATGCAAATCTTGATAAAGTTTTATCAGCATACAATAAGCATAGAGATAAGCCTATTCGTGAACAAGCCGAAAATATAATCCACTTGTTTACCTTAACTAGATTAGGTGCACCTAGAGCTTT
CAAGTATTTCGATACCACAATTGATCCAAAGCAATATCGTAGTACCAAAGAAGTTTTAGATGCTACTCTTATTCACCAAAGTATTACAGGTTTATATGAGACCCGTATTGATCTTTCTCAACTTGGT
GGTGATGAAGGTGCCCCTAAAAAGAAAAGAAAAGTTGGTTCATCATAAACTAGTTAAATAAATAAATTATTTAATAAATAATAAAAAAACAAATTGTTGTAATAATCTAATATTTTCTTTTTTTTTT
AATTTTTTTTTTTTTAAATCTTAATAATTATTAAGTTATTTTAATTTTTTTTTTTTTTTTTTTTTTTTTTTTTTTTTTTTTTCTATCAAAAAAATCAAATATATTTAAAAAATTTATTATTTACAGATACA
TTTTGAATGGTGAAGATAAATATATGCATTAGATGTAAAACAGCCAAAGAGTATGAAAATCAAAAAGATAAAGCTTAAAAAAAGCACCGACTCGGTGCCACTTTTTCAAGTTGATAACGGACTAGCC
TTATTTAAACTTGCTATGCTGTTTCCAGCATAGCTCTTAAACCGAGACGCCCGTCTCCTGCTCGATGCAGGGATCGAACCTGCGACCGTGGCGTTATTAGCACCACGCTCTGCCGACTGAGCTAATC
GAGCCTCGAGTTTTTTAAATAAAAAAATGGGTTTTTTTTAAGTAAAGTTATTTGAAATTGATTGAAATTTTCAAACCATGGGTGGTTTTTCGCTTTAAAATTGGGATTTTATTTTTATTTTTTTATAT
TTTTTATTTTTTATTTTTTTTTTTTTTGAGGTTTCTGAGATTATAAAATGAAATTTTTTTTTTTTTTTAATTAATTCAAAAAATAATCAAATAAATAAATATAATATAAAAGATCCAAAATGGATGGT
GAAGATGTTCAAGCTGGATCGTTTCGCATGATTGAACAAGATGGATTGCACGCAGGTTCTCCGGCCGCTTGGGTGGAGAGGCTATTCGGCTATGACTGGGCACAACAGACAATCGGCTGCTCTGATG
CCGCCGTGTTCCGGCTGTCAGCGCAGGGGCGCCCGGTTCTTTTTGTCAAGACCGACCTGTCCGGTGCCCTGAATGAACTGCAGGACGAGGCAGCGCGGCTATCGTGGCTGGCCACGACGGGCGTTCC
TTGCGCAGCTGTGCTCGACGTTGTCACTGAAGCGGGAAGGGACTGGCTGCTATTGGGCGAAGTGCCGGGGCAGGATCTCCTGTCATCTCACCTTGCTCCTGCCGAGAAAGTATCCATCATGGCTGAT
GCAATGCGGCGGCTGCATACGCTTGATCCGGCTACCTGCCCATTCGACCACCAAGCGAAACATCGCATCGAGCGAGCACGTACTCGGATGGAAGCCGGTCTTGTCGATCAGGATGATCTGGACGAAG
AGCATCAGGGGCTCGCGCCAGCCGAACTGTTCGCCAGGCTCAAGGCGCGCATGCCCGACGGCGAGGATCTCGTCGTGACCCATGGCGATGCCTGCTTGCCGAATATCATGGTGGAAAATGGCCGCTT
TTCTGGATTCATCGACTGTGGACGGCTGGGTGTGGCGGACCGCTATCAGGACATAGCGTTGGCTACCCGTGATATTGCTGAAGAGCTTGGCGGCGAATGGGCTGACCGCTTCCTCGTGCTTTACGGT
ATCGCCGCTCCCGATTCGCAGCGCATCGCCTTCTATCGCCTTCTTGACGAGTTCTTCTGAACTAGAATCAATTTGATTTCTTCTTAATTCAATGCTAAAAAAAAAAATATTATAAAAAAAAAAACAC
ACACAACAAATAAATTAATAAATACCATTTATTAAAAAAAAAAAAAAAAAAAAAAAAACCTATTTTAACAATTATTTTATTATTTTTATTTTTAGTTTTGTTAAAAAAAAAATTATTATCTTTATTTA
TAATTATAAAAAATTTATCCAATGATCATTTTTTAACTTTTTTATTATTTAGTATATTAAATAAAATGGATCCACTAGTTCTAGAGCGGCCGCCACCGCGGTGGAGCTCCAGCTTTTGTTCCCTTTA
GTGAGGGTTAATTGCGCGCTTGGCGTAATCATGGTCATAGCTGTTTCCTGTGTGAAATTGTTATCCGCTCACAATTCCACACAACATACGAGCCGGAAGCATAAAGTGTAAAGCCTGGGGTGCCTAA
TGAGTGAGCTAACTCACATTAATTGCGTTGCGCTCACTGCCCGCTTTCCAGTCGGGAAACCTGTCGTGCCAGCTGCATTAATGAATCGGCCAACGCGCGGGGAGAGGCGGTTTGCGTATTGGGCGCT
CTTCCGCTTCCTCGCTCACTGACTCGCTGCGCTCGGTCGTTCGGCTGCGGCGAGCGGTATCAGCTCACTCAAAGGCGGTAATACGGTTATCCACAGAATCAGGGGATAACGCAGGAAAGAACATGTG
AGCAAAAGGCCAGCAAAAGGCCAGGAACCGTAAAAAGGCCGCGTTGCTGGCGTTTTTCCATAGGCTCCGCCCCCCTGACGAGCATCACAAAAATCGACGCTCAAGTCAGAGGTGGCGAAACCCGACA
GGACTATAAAGATACCAGGCGTTTCCCCCTGGAAGCTCCCTCGTGCGCTCTCCTGTTCCGACCCTGCCGCTTACCGGATACCTGTCCGCCTTTCTCCCTTCGGGAAGCGTGGCGCTTTCTCATAGCT
CACGCTGTAGGTATCTCAGTTCGGTGTAGGTCGTTCGCTCCAAGCTGGGCTGTGTGCACGAACCCCCCGTTCAGCCCGACCGCTGCGCCTTATCCGGTAACTATCGTCTTGAGTCCAACCCGGTAAG
ACACGACTTATCGCCACTGGCAGCAGCCACTGGTAACAGGATTAGCAGAGCGAGGTATGTAGGCGGTGCTACAGAGTTCTTGAAGTGGTGGCCTAACTACGGCTACACTAGAAGGACAGTATTTGGT
ATCTGCGCTCTGCTGAAGCCAGTTACCTTCGGAAAAAGAGTTGGTAGCTCTTGATCCGGCAAACAAACCACCGCTGGTAGCGGTGGTTTTTTTGTTTGCAAGCAGCAGATTACGCGCAGAAAAAAAG
GATCTCAAGAAGATCCTTTGATCTTTTCTACGGGGTCTGACGCTCAGTGGAACGAAAACTCACGTTAAGGGATTTTGGTCATGAGATTATCAAAAAGGATCTTCACCTAGATCCTTTTAAATTAAAA
ATGAAGTTTTAAATCAATCTAAAGTATATATGAGTAAACTTGGTCTGACAGTTACCAATGCTTAATCAGTGAGGCACCTATCTCAGCGATCTGTCTATTTCGTTCATCCATAGTTGCCTGACTCCCC
GTCGTGTAGATAACTACGATACGGGAGGGCTTACCATCTGGCCCCAGTGCTGCAATGATACCGCGAGACCCACGCTCACCGGCTCCAGATTTATCAGCAATAAACCAGCCAGCCGGAAGGGCCGAGC
GCAGAAGTGGTCCTGCAACTTTATCCGCCTCCATCCAGTCTATTAATTGTTGCCGGGAAGCTAGAGTAAGTAGTTCGCCAGTTAATAGTTTGCGCAACGTTGTTGCCATTGCTACAGGCATCGTGGT
GTCACGCTCGTCGTTTGGTATGGCTTCATTCAGCTCCGGTTCCCAACGATCAAGGCGAGTTACATGATCCCCCATGTTGTGCAAAAAAGCGGTTAGCTCCTTCGGTCCTCCGATCGTTGTCAGAAGT
AAGTTGGCCGCAGTGTTATCACTCATGGTTATGGCAGCACTGCATAATTCTCTTACTGTCATGCCATCCGTAAGATGCTTTTCTGTGACTGGTGAGTACTCAACCAAGTCATTCTGAGAATAGTGTA
TGCGGCGACCGAGTTGCTCTTGCCCGGCGTCAATACGGGATAATACCGCGCCACATAGCAGAACTTTAAAAGTGCTCATCATTGGAAAACGTTCTTCGGGGCGAAAACTCTCAAGGATCTTACCGCT
GTTGAGATCCAGTTCGATGTAACCCACTCGTGCACCCAACTGATCTTCAGCATCTTTTACTTTCACCAGCGTTTCTGGGTGAGCAAAAACAGGAAGGCAAAATGCCGCAAAAAAGGGAATAAGGGCG
ACACGGAAATGTTGAATACTCATACTCTTCCTTTTTCAATATTATTGAAGCATTTATCAGGGTTATTGTCTCATGAGCGGATACATATTTGAATGTATTTAGAAAAATAAACAAATAGGGGTTCCGC
GCACATTTCCCCGAAAAGTGCCAC
```

Supplementary Figure S6. (*continued*)

```
>pTM1718(SpCas9-NG-NLS)
CTAAATTGTAAGCGTTAATATTTTGTTAAAATTCGCGTTAAATTTTTGTTAAATCAGCTCATTTTTTAACCAATAGGCCGAAATCGGCAAAATCCCTTATAAATCAAAAGAATAGACCGAGATAGGG
TTGAGTGTTGTTCCAGTTTGGAACAAGAGTCCACTATTAAAGAACGTGGACTCCAACGTCAAAGGGCGAAAAACCGTCTATCAGGGCGATGGCCCACTACGTGAACCATCACCCTAATCAAGTTTTT
TGGGGTCGAGGTGCCGTAAAGCACTAAATCGGAACCCTAAAGGGAGCCCCCGATTTAGAGCTTGACGGGGAAAGCCGGCGAACGTGGCGAGAAAGGAAGGGAAGAAAGCGAAAGGAGCGGGCGCTAG
GGCGCTGGCAAGTGTAGCGGTCACGCTGCGCGTAACCACCACACCCGCCGCGCTTAATGCGCCGCTACAGGGCGCGTCCCATTCGCCATTCAGGCTGCGCAACTGTTGGGAAGGGCGATCGGTGCGG
GCCTCTTCGCTATTACGCCAGCTGGCGAAAGGGGGATGTGCTGCAAGGCGATTAAGTTGGGTAACGCCAGGGTTTTCCCAGTCACGACGTTGTAAAACGACGGCCAGTGAGCGCGCGTAATACGACT
CACTATAGGGCGAATTGGGTACCGGGCCCCCCCTCGAGACTAGATAAAAAAAATTTTTATTTATTTTTATTTATTTTGAATTAAATAGATTACAAATTAATTAATCCCATCAAATCTTTAAAAAAAA
AAATGGTTTAAAAAACTTGGGTTGGTTAATTATTATTTGAAAATTTTAAAACCCAAATTAAAAAAAAAAAAATGGGATTCAAAAATTTTTTTTTTTTTTTTTTTTTTTTTTTTTTTTTTTCAGATTGCA
TAAAAAGATTTTTTTTTTTTTTTTTTCTTATTTCTTAAAACAAATAAATTAAATTAAATAAAAAATAAAAATCAGATCTATACAACAATAAAATGGATAAAAAGTATAGTATTGGATTAGATATCG
GTACAAATAGTGTTGGTTGGGCTGTAATAACAGATGAGTACAAGGTTCCAAGTAAGAAGTTCAAGGTATTAGGTAATACAGATAGACACAGTATTAAGAAGAATTTGATAGGTGCCTTGCTTTTTGA
TTCTGGTGAAACAGCAGAGGCCACCCGTTTGAAGAGAACAGCCAGACGTCGTTACACAAGACGTAAAAATAGAATTTGTTATTTACAAGAGATATTCAGTAATGAAATGGCCAAAGTTGATGATTCT
TTCTTCCACCGTCTTGAGGAATCTTTTTTGGTAGAAGAGGATAAAAAGCATGAAAGACACCCTATTTTCGGTAATATTGTAGATGAGGTAGCCTATCACGAAAAGTATCCTACAATCTATCACTTAA
GAAAGAAATTGGTTGATAGTACCGATAAGGCTGATCTTAGACTTATATATCTTGCACTTGCCCACATGATTAAATTCCGTGGTCATTTCTTGATCGAGGGAGATTTAAATCCAGATAATTCTGATGT
AGATAAATTGTTTATTCAATTGGTACAAACATATAATCAATTATTTGAAGAGAATCCAATCAATGCTAGTGGTGTTGATGCCAAGGCAATTCTTTCAGCCAGATTATCAAAGAGTCGTCGTTTGGAA
AATTTGATCGCACAACTTCCTGGTGAGAAGAAAAATGGATTATTTGGTAATCTTATAGCTTTGTCTTTGGGTCTTACCCCAAATTTTAAATCTAATTTTGATTTGGCAGAGGATGCTAAGTTACAAT
TGAGTAAGGATACTTACGATGATGATCTTGATAATTTGTTAGCACAAATAGGTGATCAATACGCTGATTTATTTCTTGCTGCAAAAAATTTATCTGATGCTATATTATTGTCAGATATTTTAAGAGT
TAATACTGAAATTACAAAGGCACCATTATCAGCTTCAATGATCAAGAGATACGATGAGCACCACCAAGATTTAACACTTTTAAAAGCACTTGTAAGACAACAATTGCCAGAAAAATATAAAGAAATT
TTTTTCGATCAATCAAAAAATGGTTACGCCGGATATATAGATGGTGGTGCAAGTCAAGAAGAGTTTTACAAATTTATAAAACCTATCTTAGAAAAAATGGATGGTACAGAGGAGCTTCTTGTAAAAT
TAAATCGTGAAGATTTATTACGTAAACAAAGAACCTTTGATAATGGATCTATACCTCACCAAATACACTTAGGAGAGTTACATGCTATTCTTCGTCGTCAAGAAGATTTTTATCCTTTCTTGAAAGA
TAATCGTGAGAAGATCGAGAAGATTTTAACATTCCGTATCCCTTACTATGTAGGTCCTTTGGCACGTGGTAATTCACGTTTTGCCTGGATGACAAGAAAAAGTGAGGAAACAATCACTCCTTGGAAT
TTCGAGGAGGTTGTTGATAAAGGAGCATCAGCTCAATCTTTTATAGAGAGAATGACTAATTTTGATAAGAATTTGCCAAATGAAAAAGTATTACCAAAGCACTCATTGTTATATGAGTATTTTACAG
TATACAATGAGTTGACAAAGGTAAAGTATGTTACTGAGGGTATGCGTAAACCTGCTTTTTTATCAGGTGAACAAAAGAAAGCCATAGTTGATTTACTTTTTAAGACAAATAGAAAGGTTACTGTAAA
ACAATTAAAAGAAGATTACTTTAAGAAGATCGAATGTTTCGATTCTGTTGAAATTTCAGGTGTTGAGGATCGTTTCAATGCCAGTTTAGGTACCTACCACGATTTGTTAAAGATTATCAAGGATAAG
GATTTCTTGGATAATGAGGAAAATGAGGATATTTTGGAGGATATTGTTTTGACTTTAACACTTTTCGAGGATCGTGAGATGATTGAGGAGCGTTTAAAGACCTATGCTCATCTTTTCGATGATAAAG
TTATGAAGCAATTAAAAAGACGTCGTTATACTGGATGGGGACGTTTGAGTCGTAAACTTATCAATGGTATCCGTGATAAGCAATCTGGAAAGACTATATTGGATTTTTTAAAGAGTGATGGTTTTGC
AAATCGTAATTTCATGCAACTTATTCATGATGATAGTCTTACATTCAAGGAAGATATTCAAAAAGCTCAAGTTTCAGGACAAGGAGATTCATTGCACGAACACATCGCAAATCTTGCAGGATCTCCT
GCAATTAAGAAAGGTATTTTACAAACAGTTAAAGTTGTTGATGAGCTTGTAAAGGTTATGGGTAGACATAAGCCTGAAAATATCGTTATTGAGATGGCCAGAGAAAATCAAACAACTCAAAAAGGTC
AAAAAAATTCAAGAGAGAGAATGAAAAGAATAGAGGAGGGAATTAAAGAATTAGGATCACAAATCTTGAAGGAACACCCTGTAGAAAATACTCAACTTCAAAATGAGAAACTTTACTTGTATTATCT
TCAAAATGGTCGTGATATGTATGTAGATCAAGAATTGGATATAAATCGTCTTTCAGATTATGATGTTGATCACATCGTTCCACAATCATTTTTAAAGGATGATTCAATAGATAATAAAGTTCTTACT
CGTTCTGATAAAAATCGTGGAAAATCAGATAATGTTCCATCAGAGGAGGTTGTTAAGAAAATGAAAAATTATTGGCGTCAATTGTTGAATGCCAAATTGATTACCCAAAGAAAATTTGATAATTTAA
CAAAGGCAGAACGTGGTGGATTGAGTGAGCTTGATAAAGCCGGATTCATCAAACGTCAATTGGTAGAGACTAGACAAATAACAAAACACGTAGCTCAAATTCTTGATAGTAGAATGAATACCAAATA
CGATGAAAATGATAAGTTGATAAGAGAAGTAAAAGTAATTACCCTTAAAAGTAAATTGGTATCTGATTTTCGTAAGGATTTTCAATTTTACAAAGTTAGAGAGATTAATAATTACCACCACGCCCAC
GATGCCTACTTGAATGCCGTAGTTGGTACAGCTTTGATTAAGAAATACCCTAAGTTAGAATCTGAGTTTGTATACGGTGATTACAAGGTATACGATGTAAGAAAGATGATTGCTAAATCAGAACAAG
AAATCGGTAAGGCCACTGCAAAGTACTTCTTTTATTCTAATATAATGAATTTCTTCAAAACTGAAATAACTTTGGCTAATGGAGAGATTCGTAAGCGTCCTTTAATAGAGACCAATGGAGAAACAGG
TGAGATAGTTTGGGATAAGGGACGTGATTTCGCTACTGTTCGTAAAGTTTTGTCTATGCCACAAGTTAATATCGTTAAAAAAACAGAGGTACAAACAGGTGGATTCTCAAAGGAATCAATTCGTCCT
AAGAGAAATAGTGATAAGTTAATTGCTCGTAAGAAGGATTGGGACCCAAAGAAATACGGTGGTTTCGTATCACCAACCGTAGCTTACTCAGTTTTAGTAGTAGCTAAGGTTGAAAAGGGAAAATCAA
AAAAGTTGAAATCAGTTAAGGAGTTATTAGGAATAACTATCATGGAACGTTCTTCATTCGAGAAAAATCCTATTGATTTTTTAGAGGCTAAAGGTTATAAGGAGGTAAAGAAGGATTTGATAATCAA
ATTACCTAAGTATTCTTTGTTTGAGTTAGAAAATGGACGTAAGCGTATGTTGGCAAGTGCCAGATTTTTGCAAAAGGGTAATGAACTTGCACTTCCAAGTAAGTATGTAAATTTTTTGTACTTGGCT
TCACACTACGAGAAGTTGAAGGGTTCACCAGAAGATAATGAGCAAAAGCAATTATTTGTAGAACAACACAAACATTATTTAGATGAGATTATCGAGCAAATCTCAGAGTTCTCTAAGCGTGTTATTC
TTGCTGATGCTAATTTGGATAAGGTATTATCAGCCTATAATAAGCATCGTGATAAACCAATAAGAGAACAAGCTGAGAATATTATTCACTTGTTCACTCTTACAAATTTGGGTGCCCCTAGAGCATT
TAAGTACTTCGATACAACCATTGATAGAAAGGTTTACCGTTCAACCAAAGAAGTTTTGGATGCTACTCTTATTCATCAATCTATCACAGGATTATATGAAACCCGTATAGATTTGTCTCAACTTGGT
GGTGATGAAGGTGCACCAAAGAAAAAGAGAAAGGTTGGTTCAAGTTAAACTAGTTAAATAAATAAATTATTTAATAAATAATAAAAAAACAAATTGTTGTAATAATCTAATATTTTCTTTTTTTTT
AATTTTTTTTTTTTAAATCTTAATAATTATTAAGTTATTTTAATTTTTTTTTTTTTTTTTTTTTTTTTTTTTTTTTTTTTCTATCAAAAAAATCAAATATATTTAAAAAATTTATTATTTACAGATACA
TTTTGAATGGTGAAGATAAATATATGCATTAGATGTAAAACAGCCAAAGAGTATGAAAATCAAAAAGATAAAGCTTAAAAAAAGCACCGACTCGGTGCCACTTTTTCAAGTTGATAACGGACTAGCC
TTATTTAAACTTGCTATGCTGTTTCCAGCATAGCTCTTAAACCCGTCTTCCCGAAGACCCTGCTCGATGCAGGGATCGAACCTGCGACCGTGGCGTTATTAGCACCACGCTCTGCCGACTGAGCTAA
TCGAGCCTCGAGTTTTTTAAATAAAAAATGGGTTTTTTTTAAGTAAAGTTATTTGAAATTGATTGAAATTTTCAAACCATGGGTGGTTTTTCGCTTTAAAATTGGGATTTTATTTTTATTTTTTTAT
ATTTTTTATTTTTTATTTTTTTTTTTTTGAGGTTTCTGAGATTATAAAATGAAATTTTTTTTTTTTTTTAATTAATTCAAAAAATAATCAAATAAATAAATATAATATAAAAGATCCAAAATGGATG
GTGAAGATGTTCAAGCTGGATCGTTTCGCATGATTGAACAAGATGGATTGCACGCAGGTTCTCCGGCCGCTTGGGTGGAGAGGCTATTCGGCTATGACTGGGCACAACAGACAATCGGCTGCTCTGA
TGCCGCCGTGTTCCGGCTGTCAGCGCAGGGGCGCCCGGTTCTTTTTGTCAAGACCGACCTGTCCGGTGCCCTGAATGAACTGCAGGACGAGGCAGCGCGGCTATCGTGGCTGGCCACGACGGGCGTT
CCTTGCGCAGCTGTGCTCGACGTTGTCACTGAAGCGGGAAGGGACTGGCTGCTATTGGGCGAAGTGCCGGGGCAGGATCTCCTGTCATCTCACCTTGCTCCTGCCGAGAAAGTATCCATCATGGCTG
ATGCAATGCGGCGGCTGCATACGCTTGATCCGGCTACCTGCCCATTCGACCACCAAGCGAAACATCGCATCGAGCGAGCACGTACTCGGATGGAAGCCGGTCTTGTCGATCAGGATGATCTGGACGA
AGAGCATCAGGGGCTCGCGCCAGCCGAACTGTTCGCCAGGCTCAAGGCGCGCATGCCCGACGGCGAGGATCTCGTCGTGACCCATGGCGATGCCTGCTTGCCGAATATCATGGTGGAAAATGGCCGC
TTTTCTGGATTCATCGACTGTGGACGGCTGGGTGTGGCGGACCGCTATCAGGACATAGCGTTGGCTACCCGTGATATTGCTGAAGAGCTTGGCGGCGAATGGGCTGACCGCTTCCTCGTGCTTTACG
GTATCGCCGCTCCCGATTCGCAGCGCATCGCCTTCTATCGCCTTCTTGACGAGTTCTTCTGAACTAGAATCAATTTGATTTCTTCTTAATTCAATGCTAAAAAAAAAAATATTATAAAAAAAAAAAC
ACACACAACAAATAAATTAATAAATACCATTTATTAAAAAAAAAAAAAAAAAAAAAAAAACCTATTTTAACAATTATTTTATTATTTTTATTTTTAGTTTTGTTAAAAAAAAAATTATTATCTTTATT
TATAATTATAAAAAATTTATCCAATGATCATTTTTTAACTTTTTTATTATTTAGTATATTAAATAAAATGGATCCACTAGTTCTAGAGCGGCCGCCACCGCGGTGGAGCTCCAGCTTTTGTTCCCTT
TAGTGAGGGTTAATTGCGCGCTTGGCGTAATCATGGTCATAGCTGTTTCCTGTGTGAAATTGTTATCCGCTCACAATTCCACACAACATACGAGCCGGAAGCATAAAGTGTAAAGCCTGGGGTGCCT
AATGAGTGAGCTAACTCACATTAATTGCGTTGCGCTCACTGCCCGCTTTCCAGTCGGGAAACCTGTCGTGCCAGCTGCATTAATGAATCGGCCAACGCGCGGGGAGAGGCGGTTTGCGTATTGGGCG
CTCTTCCGCTTCCTCGCTCACTGACTCGCTGCGCTCGGTCGTTCGGCTGCGGCGAGCGGTATCAGCTCACTCAAAGGCGGTAATACGGTTATCCACAGAATCAGGGGATAACGCAGGAAAGAACATG
TGAGCAAAAGGCCAGCAAAAGGCCAGGAACCGTAAAAAGGCCGCGTTGCTGGCGTTTTTCCATAGGCTCCGCCCCCCTGACGAGCATCACAAAAATCGACGCTCAAGTCAGAGGTGGCGAAACCCGA
CAGGACTATAAAGATACCAGGCGTTTCCCCCTGGAAGCTCCCTCGTGCGCTCTCCTGTTCCGACCCTGCCGCTTACCGGATACCTGTCCGCCTTTCTCCCTTCGGGAAGCGTGGCGCTTTCTCATAG
CTCACGCTGTAGGTATCTCAGTTCGGTGTAGGTCGTTCGCTCCAAGCTGGGCTGTGTGCACGAACCCCCCGTTCAGCCCGACCGCTGCGCCTTATCCGGTAACTATCGTCTTGAGTCCAACCCGGTA
AGACACGACTTATCGCCACTGGCAGCAGCCACTGGTAACAGGATTAGCAGAGCGAGGTATGTAGGCGGTGCTACAGAGTTCTTGAAGTGGTGGCCTAACTACGGCTACACTAGAAGGACAGTATTTG
GTATCTGCGCTCTGCTGAAGCCAGTTACCTTCGGAAAAAGAGTTGGTAGCTCTTGATCCGGCAAACAAACCACCGCTGGTAGCGGTGGTTTTTTTGTTTGCAAGCAGCAGATTACGCGCAGAAAAAA
AGGATCTCAAGAAGATCCTTTGATCTTTTCTACGGGGTCTGACGCTCAGTGGAACGAAAACTCACGTTAAGGGATTTTGGTCATGAGATTATCAAAAAGGATCTTCACCTAGATCCTTTTAAATTAA
AAATGAAGTTTTAAATCAATCTAAAGTATATATGAGTAAACTTGGTCTGACAGTTACCAATGCTTAATCAGTGAGGCACCTATCTCAGCGATCTGTCTATTTCGTTCATCCATAGTTGCCTGACTCC
CCGTCGTGTAGATAACTACGATACGGGAGGGCTTACCATCTGGCCCCAGTGCTGCAATGATACCGCGAGACCCACGCTCACCGGCTCCAGATTTATCAGCAATAAACCAGCCAGCCGGAAGGGCCGA
GCGCAGAAGTGGTCCTGCAACTTTATCCGCCTCCATCCAGTCTATTAATTGTTGCCGGGAAGCTAGAGTAAGTAGTTCGCCAGTTAATAGTTTGCGCAACGTTGTTGCCATTGCTACAGGCATCGTG
GTGTCACGCTCGTCGTTTGGTATGGCTTCATTCAGCTCCGGTTCCCAACGATCAAGGCGAGTTACATGATCCCCCATGTTGTGCAAAAAAGCGGTTAGCTCCTTCGGTCCTCCGATCGTTGTCAGAA
GTAAGTTGGCCGCAGTGTTATCACTCATGGTTATGGCAGCACTGCATAATTCTCTTACTGTCATGCCATCCGTAAGATGCTTTTCTGTGACTGGTGAGTACTCAACCAAGTCATTCTGAGAATAGTG
TATGCGGCGACCGAGTTGCTCTTGCCCGGCGTCAATACGGGATAATACCGCGCCACATAGCAGAACTTTAAAAGTGCTCATCATTGGAAAACGTTCTTCGGGGCGAAAACTCTCAAGGATCTTACCG
CTGTTGAGATCCAGTTCGATGTAACCCACTCGTGCACCCAACTGATCTTCAGCATCTTTTACTTTCACCAGCGTTTCTGGGTGAGCAAAAACAGGAAGGCAAAATGCCGCAAAAAAGGGAATAAGGG
CGACACGGAAATGTTGAATACTCATACTCTTCCTTTTTCAATATTATTGAAGCATTTATCAGGGTTATTGTCTCATGAGCGGATACATATTTGAATGTATTTAGAAAAATAAACAAATAGGGGTTCC
GCGCACATTTCCCCGAAAAGTGCCAC
```

Supplementary Figure S6. (*continued*)

```
>pTM1719(SpCas9-NG-NLS Esp3I)
CTAAATTGTAAGCGTTAATATTTTGTTAAAATTCGCGTTAAATTTTTGTTAAATCAGCTCATTTTTTAACCAATAGGCCGAAATCGGCAAAATCCCTTATAAATCAAAAGAATAGACCGAGATAGGG
TTGAGTGTTGTTCCAGTTTGGAACAAGAGTCCACTATTAAAGAACGTGGACTCCAACGTCAAAGGGCGAAAAACCGTCTATCAGGGCGATGGCCCACTACGTGAACCATCACCCTAATCAAGTTTTT
TGGGGTCGAGGTGCCGTAAAGCACTAAATCGGAACCCTAAAGGGAGCCCCCGATTTAGAGCTTGACGGGGAAAGCCGGCGAACGTGGCGAGAAAGGAAGGGAAGAAAGCGAAAGGAGCGGGCGCTAG
GGCGCTGGCAAGTGTAGCGGTCACGCTGCGCGTAACCACCACACCCGCCGCGCTTAATGCGCCGCTACAGGGCGCGTCCCATTCGCCATTCAGGCTGCGCAACTGTTGGGAAGGGCGATCGGTGCGG
GCCTCTTCGCTATTACGCCAGCTGGCGAAAGGGGGATGTGCTGCAAGGCGATTAAGTTGGGTAACGCCAGGGTTTTCCCAGTCACGACGTTGTAAAACGACGGCCAGTGAGCGCGCGTAATACGACT
CACTATAGGGCGAATTGGGTACCGGGCCCCCCCTCGAGACTAGATAAAAAAAATTTTTATTTATTTTTATTTATTTTGAATTAAATAGATTACAAATTAATTAATCCCATCAAATCTTTAAAAAAAA
AAATGGTTTAAAAAACTTGGGTTGGTTAATTATTATTTGAAAATTTTAAAACCCAAATTAAAAAAAAAAAAATGGGATTCAAAAATTTTTTTTTTTTTTTTTTTTTTTTTTTTTTTTTTCAGATTGCA
TAAAAAGATTTTTTTTTTTTTTTTTTTCTTATTTCTTAAAACAAATAAATTAAATTAAATAAAAAATAAAAATCAGATCTATACAACAATAAAATGGATAAAAAGTATAGTATTGGATTAGATATCG
GTACAAATAGTGTTGGTTGGGCTGTAATAACAGATGAGTACAAGGTTCCAAGTAAGAAGTTCAAGGTATTAGGTAATACAGATAGACACAGTATTAAGAAGAATTTGATAGGTGCCTTGCTTTTTGA
TTCTGGTGAAACAGCAGAGGCCACCCGTTTGAAGAGAACAGCCAGACGTCGTTACACAAGACGTAAAAATAGAATTTGTTATTTACAAGAGATATTCAGTAATGAAATGGCCAAAGTTGATGATTCT
TTCTTCCACCGTCTTGAGGAATCTTTTTTGGTAGAAGAGGATAAAAAGCATGAAAGACACCCTATTTTCGGTAATATTGTAGATGAGGTAGCCTATCACGAAAAGTATCCTACAATCTATCACTTAA
GAAAGAAATTGGTTGATAGTACCGATAAGGCTGATCTTAGACTTATATATCTTGCACTTGCCCACATGATTAAATTCCGTGGTCATTTCTTGATCGAGGGAGATTTAAATCCAGATAATTCTGATGT
AGATAAATTGTTTATTCAATTGGTACAAACATATAATCAATTATTTGAAGAGAATCCAATCAATGCTAGTGGTGTTGATGCCAAGGCAATTCTTTCAGCCAGATTATCAAAGAGTCGTCGTTTGGAA
AATTTGATCGCACAACTTCCTGGTGAGAAGAAAAATGGATTATTTGGTAATCTTATAGCTTTGTCTTTGGGTCTTACCCCAAATTTTAAATCTAATTTTGATTTGGCAGAGGATGCTAAGTTACAAT
TGAGTAAGGATACTTACGATGATGATCTTGATAATTTGTTAGCACAAATAGGTGATCAATACGCTGATTTATTTCTTGCTGCAAAAAATTTATCTGATGCTATATTATTGTCAGATATTTTAAGAGT
TAATACTGAAATTACAAAGGCACCATTATCAGCTTCAATGATCAAGAGATACGATGAGCACCACCAAGATTTAACACTTTTAAAAGCACTTGTAAGACAACAATTGCCAGAAAAATATAAAGAAATT
TTTTTCGATCAATCAAAAAATGGTTACGCCGGATATATAGATGGTGGTGCAAGTCAAGAAGAGTTTTACAAATTTATAAAACCTATCTTAGAAAAAATGGATGGTACAGAGGAGCTTCTTGTAAAAT
TAAATCGTGAAGATTTATTACGTAAACAAAGAACCTTTGATAATGGATCTATACCTCACCAAATACACTTAGGAGAGTTACATGCTATTCTTCGTCGTCAAGAAGATTTTTATCCTTTCTTGAAAGA
TAATCGTGAGAAGATCGAGAAGATTTTAACATTCCGTATCCCTTACTATGTAGGTCCTTTGGCACGTGGTAATTCACGTTTTGCCTGGATGACAAGAAAAAGTGAGGAAACAATCACTCCTTGGAAT
TTCGAGGAGGTTGTTGATAAAGGAGCATCAGCTCAATCTTTTATAGAGAGAATGACTAATTTTGATAAGAATTTGCCAAATGAAAAAGTATTACCAAAGCACTCATTGTTATATGAGTATTTTACAG
TATACAATGAGTTGACAAAGGTAAAGTATGTTACTGAGGGTATGCGTAAACCTGCTTTTTTATCAGGTGAACAAAAGAAAGCCATAGTTGATTTACTTTTTAAGACAAATAGAAAGGTTACTGTAAA
ACAATTAAAAGAAGATTACTTTAAGAAGATCGAATGTTTCGATTCTGTTGAAATTTCAGGTGTTGAGGATCGTTTCAATGCCAGTTTAGGTACCTACCACGATTTGTTAAAGATTATCAAGGATAAG
GATTTCTTGGATAATGAGGAAAATGAGGATATTTTGGAGGATATTGTTTTGACTTTAACACTTTTCGAGGATCGTGAGATGATTGAGGAGCGTTTAAAGACCTATGCTCATCTTTTCGATGATAAAG
TTATGAAGCAATTAAAAAGACGTCGTTATACTGGATGGGGACGTTTGAGTCGTAAACTTATCAATGGTATCCGTGATAAGCAATCTGGAAAGACTATATTGGATTTTTTAAAGAGTGATGGTTTTGC
AAATCGTAATTTCATGCAACTTATTCATGATGATAGTCTTACATTCAAGGAAGATATTCAAAAAGCTCAAGTTTCAGGACAAGGAGATTCATTGCACGAACACATCGCAAATCTTGCAGGATCTCCT
GCAATTAAGAAAGGTATTTTACAAACAGTTAAAGTTGTTGATGAGCTTGTAAAGGTTATGGGTAGACATAAGCCTGAAAATATCGTTATTGAGATGGCCAGAGAAAATCAAACAACTCAAAAAGGTC
AAAAAAATTCAAGAGAGAGAATGAAAAGAATAGAGGAGGGAATTAAAGAATTAGGATCACAAATCTTGAAGGAACACCCTGTAGAAAATACTCAACTTCAAAATGAGAAACTTTACTTGTATTATCT
TCAAAATGGTCGTGATATGTATGTAGATCAAGAATTGGATATAAATCGTCTTTCAGATTATGATGTTGATCACATCGTTCCACAATCATTTTTAAAGGATGATTCAATAGATAATAAAGTTCTTACT
CGTTCTGATAAAAATCGTGGAAAATCAGATAATGTTCCATCAGAGGAGGTTGTTAAGAAAATGAAAAATTATTGGCGTCAATTGTTGAATGCCAAATTGATTACCCAAAGAAAATTTGATAATTTAA
CAAAGGCAGAACGTGGTGGATTGAGTGAGCTTGATAAAGCCGGATTCATCAAACGTCAATTGGTAGAGACTAGACAAATAACAAAACACGTAGCTCAAATTCTTGATAGTAGAATGAATACCAAATA
CGATGAAAATGATAAGTTGATAAGAGAAGTAAAAGTAATTACCCTTAAAAGTAAATTGGTATCTGATTTTCGTAAGGATTTTCAATTTTACAAAGTTAGAGAGATTAATAATTACCACCACGCCCAC
GATGCCTACTTGAATGCCGTAGTTGGTACAGCTTTGATTAAGAAATACCCTAAGTTAGAATCTGAGTTTGTATACGGTGATTACAAGGTATACGATGTAAGAAAGATGATTGCTAAATCAGAACAAG
AAATCGGTAAGGCCACTGCAAAGTACTTCTTTTATTCTAATATAATGAATTTCTTCAAAACTGAAATAACTTTGGCTAATGGAGAGATTCGTAAGCGTCCTTTAATAGAGACCAATGGAGAAACAGG
TGAGATAGTTTGGGATAAGGGACGTGATTTCGCTACTGTTCGTAAAGTTTTGTCTATGCCACAAGTTAATATCGTTAAAAAAACAGAGGTACAAACAGGTGGATTCTCAAAGGAATCAATTCGTCCT
AAGAGAAATAGTGATAAGTTAATTGCTCGTAAGAAGGATTGGGACCCAAAGAAATACGGTGGTTTCGTATCACCAACCGTAGCTTACTCAGTTTTAGTAGTAGCTAAGGTTGAAAAGGGAAAATCAA
AAAAGTTGAAATCAGTTAAGGAGTTATTAGGAATAACTATCATGGAACGTTCTTCATTCGAGAAAAATCCTATTGATTTTTTAGAGGCTAAAGGTTATAAGGAGGTAAAGAAGGATTTGATAATCAA
ATTACCTAAGTATTCTTTGTTTGAGTTAGAAAATGGACGTAAGCGTATGTTGGCAAGTGCCAGATTTTTGCAAAAGGGTAATGAACTTGCACTTCCAAGTAAGTATGTAAATTTTTTGTACTTGGCT
TCACACTACGAGAAGTTGAAGGGTTCACCAGAAGATAATGAGCAAAAGCAATTATTTGTAGAACAACACAAACATTATTTAGATGAGATTATCGAGCAAATCTCAGAGTTCTCTAAGCGTGTTATTC
TTGCTGATGCTAATTTGGATAAGGTATTATCAGCCTATAATAAGCATCGTGATAAACCAATAAGAGAACAAGCTGAGAATATTATTCACTTGTTCACTCTTACAAATTTGGGTGCCCCTAGAGCATT
TAAGTACTTCGATACAACCATTGATAGAAAGGTTTACCGTTCAACCAAAGAAGTTTTGGATGCTACTCTTATTCATCAATCTATCACAGGATTATATGAAACCCGTATAGATTTGTCTCAACTTGGT
GGTGATGAAGGTGCACCAAAGAAAAAGAGAAAGGTTGGTTCAAGTTAAACTAGTTAAATAAATAAATTATTTAATAAATAATAAAAAAACAAATTGTTGTAATAATCTAATATTTTCTTTTTTTTTT
AATTTTTTTTTTTTTAAATCTTAATAATTATTAAGTTATTTTAATTTTTTTTTTTTTTTTTTTTTTTTTTTTTTTTTTTTTCTATCAAAAAAATCAAATATATTTAAAAAATTTATTATTTACAGATACA
TTTTGAATGGTGAAGATAAATATATGCATTAGATGTAAAACAGCCAAAGAGTATGAAAATCAAAAAGATAAAGCTTAAAAAAAGCACCGACTCGGTGCCACTTTTTCAAGTTGATAACGGACTAGCC
TTATTTAAACTTGCTATGCTGTTTCCAGCATAGCTCTTAAACCGAGACGCCCGTCTCCTGCTCGATGCAGGGATCGAACCTGCGACCGTGGCGTTATTAGCACCACGCTCTGCCGACTGAGCTAATC
GAGCCTCGAGTTTTTTAAATAAAAAAATGGGTTTTTTTTAAGTAAAGTTATTTGAAATTGATTGAAATTTTCAAACCATGGGTGGTTTTTCGCTTTAAAATTGGGATTTTATTTTTATTTTTTTATAT
TTTTTATTTTTTATTTTTTTTTTTTTGAGGTTTCTGAGATTATAAAATGAAATTTTTTTTTTTTTTTAATTAATTCAAAAAATAATCAAATAAATAAATATAATATAAAAGATCCAAAATGGATGGT
GAAGATGTTCAAGCTGGATCGTTTCGCATGATTGAACAAGATGGATTGCACGCAGGTTCTCCGGCCGCTTGGGTGGAGAGGCTATTCGGCTATGACTGGGCACAACAGACAATCGGCTGCTCTGATG
CCGCCGTGTTCCGGCTGTCAGCGCAGGGGCGCCCGGTTCTTTTTGTCAAGACCGACCTGTCCGGTGCCCTGAATGAACTGCAGGACGAGGCAGCGCGGCTATCGTGGCTGGCCACGACGGGCGTTCC
TTGCGCAGCTGTGCTCGACGTTGTCACTGAAGCGGGAAGGGACTGGCTGCTATTGGGCGAAGTGCCGGGGCAGGATCTCCTGTCATCTCACCTTGCTCCTGCCGAGAAAGTATCCATCATGGCTGAT
GCAATGCGGCGGCTGCATACGCTTGATCCGGCTACCTGCCCATTCGACCACCAAGCGAAACATCGCATCGAGCGAGCACGTACTCGGATGGAAGCCGGTCTTGTCGATCAGGATGATCTGGACGAAG
AGCATCAGGGGCTCGCGCCAGCCGAACTGTTCGCCAGGCTCAAGGCGCGCATGCCCGACGGCGAGGATCTCGTCGTGACCCATGGCGATGCCTGCTTGCCGAATATCATGGTGGAAAATGGCCGCTT
TTCTGGATTCATCGACTGTGGACGGCTGGGTGTGGCGGACCGCTATCAGGACATAGCGTTGGCTACCCGTGATATTGCTGAAGAGCTTGGCGGCGAATGGGCTGACCGCTTCCTCGTGCTTTACGGT
ATCGCCGCTCCCGATTCGCAGCGCATCGCCTTCTATCGCCTTCTTGACGAGTTCTTCTGAACTAGAATCAATTTGATTTCTTCTTAATTCAATGCTAAAAAAAAAAATATTATAAAAAAAAAAACAC
ACACAACAAATAAATTAATAAATACCATTTATTAAAAAAAAAAAAAAAAAAAAAAAAACCTATTTTAACAATTATTTTATTATTTTTATTTTTAGTTTTGTTAAAAAAAAAATTATTATCTTTATTTA
TAATTATAAAAAATTTATCCAATGATCATTTTTTAACTTTTTTATTATTTAGTATATTAAATAAAATGGATCCACTAGTTCTAGAGCGGCCGCCACCGCGGTGGAGCTCCAGCTTTTGTTCCCTTTA
GTGAGGGTTAATTGCGCGCTTGGCGTAATCATGGTCATAGCTGTTTCCTGTGTGAAATTGTTATCCGCTCACAATTCCACACAACATACGAGCCGGAAGCATAAAGTGTAAAGCCTGGGGTGCCTAA
TGAGTGAGCTAACTCACATTAATTGCGTTGCGCTCACTGCCCGCTTTCCAGTCGGGAAACCTGTCGTGCCAGCTGCATTAATGAATCGGCCAACGCGCGGGGAGAGGCGGTTTGCGTATTGGGCGCT
CTTCCGCTTCCTCGCTCACTGACTCGCTGCGCTCGGTCGTTCGGCTGCGGCGAGCGGTATCAGCTCACTCAAAGGCGGTAATACGGTTATCCACAGAATCAGGGGATAACGCAGGAAAGAACATGTG
AGCAAAAGGCCAGCAAAAGGCCAGGAACCGTAAAAAGGCCGCGTTGCTGGCGTTTTTCCATAGGCTCCGCCCCCCTGACGAGCATCACAAAAATCGACGCTCAAGTCAGAGGTGGCGAAACCCGACA
GGACTATAAAGATACCAGGCGTTTCCCCCTGGAAGCTCCCTCGTGCGCTCTCCTGTTCCGACCCTGCCGCTTACCGGATACCTGTCCGCCTTTCTCCCTTCGGGAAGCGTGGCGCTTTCTCATAGCT
CACGCTGTAGGTATCTCAGTTCGGTGTAGGTCGTTCGCTCCAAGCTGGGCTGTGTGCACGAACCCCCCGTTCAGCCCGACCGCTGCGCCTTATCCGGTAACTATCGTCTTGAGTCCAACCCGGTAAG
ACACGACTTATCGCCACTGGCAGCAGCCACTGGTAACAGGATTAGCAGAGCGAGGTATGTAGGCGGTGCTACAGAGTTCTTGAAGTGGTGGCCTAACTACGGCTACACTAGAAGGACAGTATTTGGT
ATCTGCGCTCTGCTGAAGCCAGTTACCTTCGGAAAAAGAGTTGGTAGCTCTTGATCCGGCAAACAAACCACCGCTGGTAGCGGTGGTTTTTTTGTTTGCAAGCAGCAGATTACGCGCAGAAAAAAAG
GATCTCAAGAAGATCCTTTGATCTTTTCTACGGGGTCTGACGCTCAGTGGAACGAAAACTCACGTTAAGGGATTTTGGTCATGAGATTATCAAAAAGGATCTTCACCTAGATCCTTTTAAATTAAAA
ATGAAGTTTTAAATCAATCTAAAGTATATATGAGTAAACTTGGTCTGACAGTTACCAATGCTTAATCAGTGAGGCACCTATCTCAGCGATCTGTCTATTTCGTTCATCCATAGTTGCCTGACTCCCC
GTCGTGTAGATAACTACGATACGGGAGGGCTTACCATCTGGCCCCAGTGCTGCAATGATACCGCGAGACCCACGCTCACCGGCTCCAGATTTATCAGCAATAAACCAGCCAGCCGGAAGGGCCGAGC
GCAGAAGTGGTCCTGCAACTTTATCCGCCTCCATCCAGTCTATTAATTGTTGCCGGGAAGCTAGAGTAAGTAGTTCGCCAGTTAATAGTTTGCGCAACGTTGTTGCCATTGCTACAGGCATCGTGGT
GTCACGCTCGTCGTTTGGTATGGCTTCATTCAGCTCCGGTTCCCAACGATCAAGGCGAGTTACATGATCCCCCATGTTGTGCAAAAAAGCGGTTAGCTCCTTCGGTCCTCCGATCGTTGTCAGAAGT
AAGTTGGCCGCAGTGTTATCACTCATGGTTATGGCAGCACTGCATAATTCTCTTACTGTCATGCCATCCGTAAGATGCTTTTCTGTGACTGGTGAGTACTCAACCAAGTCATTCTGAGAATAGTGTA
TGCGGCGACCGAGTTGCTCTTGCCCGGCGTCAATACGGGATAATACCGCGCCACATAGCAGAACTTTAAAAGTGCTCATCATTGGAAAACGTTCTTCGGGGCGAAAACTCTCAAGGATCTTACCGCT
GTTGAGATCCAGTTCGATGTAACCCACTCGTGCACCCAACTGATCTTCAGCATCTTTTACTTTCACCAGCGTTTCTGGGTGAGCAAAAACAGGAAGGCAAAATGCCGCAAAAAAGGGAATAAGGGCG
ACACGGAAATGTTGAATACTCATACTCTTCCTTTTTCAATATTATTGAAGCATTTATCAGGGTTATTGTCTCATGAGCGGATACATATTTGAATGTATTTAGAAAAATAAACAAATAGGGGTTCCGC
GCACATTTCCCCGAAAAGTGCCAC
```

Supplementary Figure S6. Predicted DNA sequences of the all-in-one vectors.

Supplementary Table S1. List of oligonucleotides used to generate the all-in-one targeting constructs.

Target gene	PAM	Sequence (5'- to -3') Forward	Sequence (5'- to -3') Reverse
tdTomato	NAA	agcaGGGGACAGGATGTCCCAGGC	aaacGCCTGGGACATCCTGTCCCC
tdTomato	NAT	agcaTCGGTGGAGGCCTCCCAGCC	aaacGGCTGGGAGGCCTCCACCGA
tdTomato	NAG	agcaGAGTCCTGGGTCACGGTCAC	aaacGTGACCGTGACCCAGGACTC
tdTomato	NAC	agcaTGGTGGATCTCGCCCTTCAG	aaacCTGAAGGGCGAGATCCACCA
tdTomato	NTA	agcaGTGCCGCGCATCTTCACCTT	aaacAAGGTGAAGATGCGCGGCAC
tdTomato	NTT	agcaACGGGGCCGTCGGGGGGGAA	aaacTTCCCCCCCGACGGCCCCGT
tdTomato	NTG	agcaCCGTCCTGCAGGGAGGAGTC	aaacGACTCCTCCCTGCAGGACGG
tdTomato	NTC	agcaCCGTCGCGGGGGTACAGGCG	aaacCGCCTGTACCCCCGCGACGG
tdTomato	NGA	agcaGGGTGCTTCACGTACGCCTT	aaacAAGGCGTACGTGAAGCACCC
tdTomato	NGT	agcaCTTCACGTACGCCTTGGAGC	aaacGCTCCAAGGCGTACGTGAAG
tdTomato	NGG	agcaATGTCCCAGGCGAAGGGCAG	aaacCTGCCCTTCGCCTGGGACAT
tdTomato	NGC	agcaTTCTTGTAATCGGGGATGTC	aaacGACATCCCCGATTACAAGAA
tdTomato	NCA	agcaGTAGATGGTCTTGAACTCCA	aaacTGGAGTTCAAGACCATCTAC
tdTomato	NCT	agcaGCCGTCCTTCAGCTTCAGGG	aaacCCCTGAAGCTGAAGGACGGC
tdTomato	NCG	agcaGGGTCACGGTCACCAGACCG	aaacCGGTCTGGTGACCGTGACCC
tdTomato	NCC	agcaGGTACAGGCGCTCGGTGGAG	aaacCTCCACCGAGCGCCTGTACC
carA	NAT	agcaGGATTTCCATCTAAAAGACC	aaacGGTCTTTTAGATGGAAATCC
carA	NTA	agcaTTCCATCTAAAAGACCCATT	aaacAATGGGTCTTTTAGATGGAA
carA	NGA	agcaTTAGATGGAAATCCAGCCAA	aaacTTGGCTGGATTTCCATCTAA
H3a	NAC	agcaAAGTGGTGGTGTAAAGAAAG	aaacCTTTCTTTACACCACCACTT
H3a	NGA	agcaTTCTTTACACCACCACTTGA	aaacTCAAGTGGTGGTGTAAAGAA
H2Bv3	NGC	agcaCAATCTGGTGAAGAGAAAAC	aaacGTTTTCTCTTCACCAGATTG

Upper- and lower-case letters indicate the target sequence and overhangs for Golden Gate assembly, respectively.

Supplementary Table S2. All-in-one vectors for Cas9 variants.

all-in-one vector	Cas9	GFP	Restriction enzyme
pTM1285	SpCas9	+	BpiI
pTM1416	SpCas9	+	Esp3I
pTM1593	xCas9 3.7	-	BpiI
pTM1718	SpCas9-NG	-	BpiI
pTM1719	SpCas9-NG	-	Esp3I
pTM1668	SpRY	-	Esp3I
pTM1599	SpCas9	-	BpiI
pTM1644	SpCas9	-	Esp3I
pTM1544	Cas9 nickase	+	BpiI

Restriction enzymes used for Golden Gate assembly are shown in the far right column.

Supplementary Table S3. Targeting constructs expressing various Cas9 and sgRNAs.

Plasmid	Backbone vector	Cas9 Type	Target Gene	PAM
pTM1519	pTM1285	SpCas9	*tdTomato*	NGA
pTM1518	pTM1285	SpCas9	*tdTomato*	NGT
pTM1517	pTM1285	SpCas9	*tdTomato*	NGG
pTM1516	pTM1285	SpCas9	*tdTomato*	NGC
pTM1620	pTM1416	SpCas9	*tdTomato*	NGC
pTM1621	pTM1599	SpCas9	*tdTomato*	NGG
pTM1647	pTM1644	SpCas9	*tdTomato*	NGG
pTM1612	pTM1593	xCas9 3.7	*tdTomato*	NGA
pTM1613	pTM1593	xCas9 3.7	*tdTomato*	NGT
pTM1614	pTM1593	xCas9 3.7	*tdTomato*	NGG
pTM1615	pTM1593	xCas9 3.7	*tdTomato*	NGC
pTM1616	pTM1718	SpCas9-NG	*tdTomato*	NGA
pTM1617	pTM1718	SpCas9-NG	*tdTomato*	NGT
pTM1618	pTM1718	SpCas9-NG	*tdTomato*	NGG
pTM1619	pTM1718	SpCas9-NG	*tdTomato*	NGC
pTM1636	pTM1719	SpCas9-NG	*tdTomato*	NGG
pTM1671	pTM1668	SpRY	*tdTomato*	NGA
pTM1672	pTM1668	SpRY	*tdTomato*	NGT
pTM1673	pTM1668	SpRY	*tdTomato*	NGG
pTM1674	pTM1668	SpRY	*tdTomato*	NGC
pTM1679	pTM1668	SpRY	*tdTomato*	NAA
pTM1680	pTM1668	SpRY	*tdTomato*	NAT
pTM1681	pTM1668	SpRY	*tdTomato*	NAG
pTM1682	pTM1668	SpRY	*tdTomato*	NAC
pTM1683	pTM1668	SpRY	*tdTomato*	NTA
pTM1684	pTM1668	SpRY	*tdTomato*	NTT
pTM1685	pTM1668	SpRY	*tdTomato*	NTG
pTM1686	pTM1668	SpRY	*tdTomato*	NTC
pTM1687	pTM1668	SpRY	*tdTomato*	NCA
pTM1688	pTM1668	SpRY	*tdTomato*	NCT
pTM1689	pTM1668	SpRY	*tdTomato*	NCG
pTM1690	pTM1668	SpRY	*tdTomato*	NCC
pTM1735	pTM1285	SpCas9	*carA*	NAT
pTM1712	pTM1285	SpCas9	*carA*	NTA
pTM1713	pTM1285	SpCas9	*carA*	NGA
pTM1737	pTM1718	SpCas9-NG	*carA*	NAT
pTM1720	pTM1718	SpCas9-NG	*carA*	NTA
pTM1721	pTM1718	SpCas9-NG	*carA*	NGA
pTM1746	pTM1668	SpRY	*carA*	NAT
pTM1715	pTM1668	SpRY	*carA*	NTA
pTM1716	pTM1668	SpRY	*carA*	NGA
pTM1554	pTM1544	Cas9 nickase	*H2Bv3*	NGG
pTM1743	pTM1668	SpRY	*H2Bv3*	NGC
pTM1553	pTM1544	Cas9 nickase	*H3a*	NGG
pTM1741	pTM1668	SpRY	*H3a*	NGA
pTM1742	pTM1668	SpRY	*H3a*	NAC
pTM1745	pTM1718	SpCas9-NG	*H3a*	NGA

The PAM columns show the PAM sequences following the target sequences.

Supplementary Table S4. List of primers used in this study.

Name	Sequence (5'- to -3')
tracr-Rv	AAGCTTAAAAAAAGCACCGACTCGGTGCC
NeoUp	TCCTGCAGTTCATTCAGGGC
NeoUp2	CTTCCCGCTTCAGTGACAAC
GFPdown	TGGAAGCGTTCAACTAGCAG
GFPcAR1-arm(60bp) Fw	acatacataaactatctagatttttcacacatatatatatataaataaaaaaataaaatgAGTAAAGGAGAAGAACTTTTCACTG
GFPcAR1-arm(60bp) Rv	atcggcaaataataataaaaccaatgatgtttcattggctggatttccatctaaaagaccTTTGTATAGTTCATCCATGCCATG
GFPcAR1-arm(30bp) Fw	atatatatatataaataaaaaaataaaatgAGTAAAGGAGAAGAACTTTTCACTG
GFPcAR1-arm(30bp) Rv	ttcattggctggatttccatctaaaagaccTTTGTATAGTTCATCCATGCCATG
GFPcAR1-arm(90bp) Fw	aaaaaaaaaaaaaattatttaaaatacaatcacatacataaactatctagatttttcacacatatatatatataaataaaaaaataaaatgAGTAAAGGAGAAGAACTTTTCACTG
GFPcAR1-arm(90bp) Rv	cactgccatacaacccaacattgaagaaaaatcggcaaataataataaaaccaatgatgtttcattggctggatttccatctaaaagaccTTTGTATAGTTCATCCATGCCATG
carA-forSeq-Fw	TCGTTCACCACCACTACACT
carA-forSeq-Rv	CAACACTGCCATACAACCCA
carA-forSeq-Rv2	GGAAACCACCATTGACAGCT
H3a K39A mutation detection-Fw	CATCAAGTGGTGGTGTAgcc
H3a Rv	TCACCTCTGATACGTCTGGC
H3a-forSeq-Fw	CGTACAAAACAAACCGCACG
H2Bv3 E18AE19A mutation detection-Fw	CAACTCAATCTGGTgccgct
H2Bv3 Rv	ATGATACGAACGGCAACTTG
H2Bv3-forSeq-Fw	TCTCCAACACATTCCAATCA

Supplementary Table S5. List of oligonucleotides used to generate Cas9-nickase constructs.

Target gene	Direction	Sequence (5'- to -3') Forward	Sequence (5'- to -3') Reverse
H3a	+	gagcaAAAGTACATCGTTTCAGACCg	taaacGGTCTGAAACGATGTACTTTt
H3a	-	agcaCCACCACTTGATGAAGAGAC	aaacGTCTCTTCATCAAGTGGTGG
H2Bv3	+	gagcaGTAACCAAAACCCCAACTGAg	taaacTCAGTTGGGGTTTTGGTTACt
H2Bv3	-	agcaACCAGATTGAGTTGAACCTT	aaacAAGGTTCAACTCAATCTGGT

Upper- and lower-case letters indicate the target sequence and overhangs for Golden Gate assembly, respectively.
